# Hepatokine ORM2 suppresses pathological ferritinophagy to prevent acute tissue injury

**DOI:** 10.1038/s41419-026-08803-0

**Published:** 2026-04-30

**Authors:** Hongyi Tang, Shu Wang, Fang Liu, Boqian Shen, Feng Jiang, Xinyuan Xiong, Nan Yang, Huiqin Zhu, Rulin Zhang, Dongge Xia, Cong Cong, Yan Lu, Jie Yang, Jun Wu, Bing Zhou, Xuxu Sun

**Affiliations:** 1 Department of Biochemistry and Molecular Cell Biology, State Key Laboratory of Systems Medicine for Cancer, Shanghai Key Laboratory for Tumor Microenvironment and Inflammation, Jiading Branch of Shanghai General Hospital, Shanghai Jiao Tong University School of Medicine, Shanghai, China; 2 Department of Laboratory Medicine, Jiading Branch of Shanghai General Hospital, Shanghai Jiao Tong University School of Medicine, Shanghai, China; 3 Department of Laboratory Medicine, Shanghai General Hospital, Shanghai Jiao Tong University School of Medicine, Shanghai, China; 4 Cardiovascular Department, Affiliated Hospital of Shandong University of Traditional Chinese Medicine, Jinan, Shandong China; 5 Institute of Metabolism and Regenerative Medicine, Digestive Endoscopic Center, Shanghai Sixth People’s Hospital Affiliated to Shanghai Jiao Tong University School of Medicine, Shanghai, China; 6 Department of Pathology, The Affiliated Hospital of Youjiang Medical University for Nationalities; Key Laboratory of Molecular Pathology in Tumors of Guangxi Higher Education Institutes, Baise, China; 7 Shanghai Diabetes Institute, Shanghai Key Laboratory of Diabetes Mellitus, Shanghai Clinical Centre for Diabetes, Shanghai Sixth People’s Hospital Affiliated to Shanghai Jiao Tong University School of Medicine, Shanghai, China

**Keywords:** Cell signalling, Gastrointestinal diseases

## Abstract

Acute tissue injuries trigger rapid cellular damage, but cell-intrinsic protective programs attenuate pathology through regulatory axes. Here, we demonstrate that Orosomucoid 2 (ORM2), a hepatokine, is significantly upregulated during drug-induced acute liver injury. Knock out *Orm2* exacerbates liver damage, while its overexpression provides protection, identifying ORM2 as an endogenous hepatoprotective factor during acute liver injury. Mechanistically, ORM2 disrupts the FTH-NCOA4-TAX1BP1 interaction, thereby blocking ferritinophagy and preventing iron overload-induced cytotoxicity. Furthermore, acute ischemia-reperfusion injuries in other organs also trigger hepatic ORM2 upregulation and secretion, demonstrating liver-organ crosstalk via the circulatory system. Exogenous administration of ORM2 effectively ameliorates ferroptosis and tissue damage in multiple ischemia-reperfusion injury models. Collectively, our results identify the hepatokine ORM2 as a key suppressor of pathological ferritinophagy. This function positions ORM2 protein as a potential therapeutic candidate for acute tissue injuries driven by ferritinophagy-mediated iron overload.

## Introduction

The cellular stress response is a mechanism by which cells respond to and protect against stress-induced disruptions in homeostasis. Investigating the key factors and signaling pathways involved in cellular defense and protection is important for understanding disease pathophysiology and identifying potential therapeutic targets in the clinic.

Acute liver injury, whether from drug-induced liver injury (DILI) or hepatic ischemia followed by reperfusion (I/R), provokes massive hepatocyte death and creates a hypoxic-ischemic microenvironment that can progress to liver dysfunction, acute liver failure and even mortality [1–4]. Hepatic cell death represents a hallmark feature of acute liver injury, with pathways such as apoptosis, necrosis, ferroptosis, pyroptosis, and necroptosis [5–10]. Ferroptosis, a unique form of non-apoptotic cell death, is characterized by the accumulation of iron. Excess iron catalyzes the conversion of hydrogen peroxide (H_2_O_2_) through Fenton Reaction, generating ROS and inducing lipid peroxidation and cell death [8, 11–13]. The pathological significance of ferroptosis is now recognized across a remarkably broad spectrum of diseases [14–19]. The role of iron overload and dysregulated ferrous-ion homeostasis is particularly well-delineated in the context of hepatic pathologies, including ischemia-reperfusion and drug-induced acute liver injury [8, 20–22]. Intervening in these pathways using the iron chelator deferoxamine mesylate (DFO) or other specific inhibitors, consistently mitigates hepatocyte death and protects against acute liver injury, underscoring the pivotal role of iron homeostasis in dictating the extent of liver damage [23–27].

Hepatokines are a group of liver-specific secretory proteins that act via paracrine signaling on neighboring cells or endocrine signaling on distant organs. Among these, acute phase response proteins (APRs), such as C-reactive protein, Orosomucoid (ORM), and serum amyloid A (SAA), are a class of proteins that are rapidly upregulated, predominantly synthesized and secreted by the liver in response to tissue injury, infection or malignancy [28]. ORM, also known as alpha-1 acid glycoprotein, is an acute-phase protein induced by cytokines such as IL-1β, TNF-α, and IL-6 during inflammatory responses and tissue injury, including DILI [29, 30]. The ORM family comprises two variants (ORM1 and ORM2) in humans and three variants (ORM1, ORM2, and ORM3) in mice [31]. Under basal conditions, ORM1 is primarily secreted by hepatocytes and adipocytes, constituting approximately 75% of circulating ORMs and functioning primarily in modulating adipose tissue inflammation, insulin sensitivity, and appetite. In contrast, ORM2 is highly induced under metabolic stress (e.g., fatty liver) and acute injury, with its core functions centered on regulating hepatic lipid homeostasis and inflammatory responses [32]. The function of mouse-specific ORM3 is not well characterized, and it is expressed at low basal levels. Accumulating evidence has established that ORM expression levels correlate with disease progression and may serve as valuable biomarkers for prognosis in various pathological conditions, such as malignancies and rheumatoid arthritis [31, 33–35]. Functionally, ORMs have been shown to participate in diverse biological processes, including immunomodulation, protein transport, and lipid metabolism [29, 36–38]. In the context of acute injury, ORM2 has been implicated in post-stroke recovery processes, and exogenous ORM administration has been demonstrated to protect against renal ischemia-reperfusion (I/R) injury [39, 40]. However, the pathophysiological significance of rapid ORM induction and its detailed molecular mechanisms during the acute phase response to tissue injury remains poorly understood.

In this study, we demonstrated that ORM2 expression and secretion were rapidly induced in Acetaminophen (APAP)-treated livers. Genetic ablation of *Orm2* exacerbated APAP-induced liver damage, indicating its protective function in the local microenvironment. Mechanistically, we revealed that ORM2 suppressed ferritinophagy by disrupting the formation of the TAX1BP1-NCOA4-ferritin complex, thereby attenuating ferrous ion overload and its associated toxicity. Our data establish ORM2 as a novel regulator of iron metabolism under conditions of APAP-induced hepatotoxic stress. We further demonstrated that hepatic ORM2 secretion was induced not only under liver injury but also in response to ischemia-reperfusion injuries in various distant organs, and that circulating ORM2 exerts protective effects in those tissues. Importantly, treatment with recombinant ORM2 protein reduced ferroptosis-mediated tissue damage across multiple ischemia-reperfusion models. Collectively, our findings uncover a molecular mechanism by which hepatocytes coordinate a protective response against both local and systemic acute injury. These results highlight the therapeutic potential of ORM2 in mitigating ferrotinophagy-driven ferrous ion toxicity and tissue damage across diverse organ systems.

## Results

### Loss of *Orm2* exacerbates APAP-induced acute liver injury

**Fig. 1:**
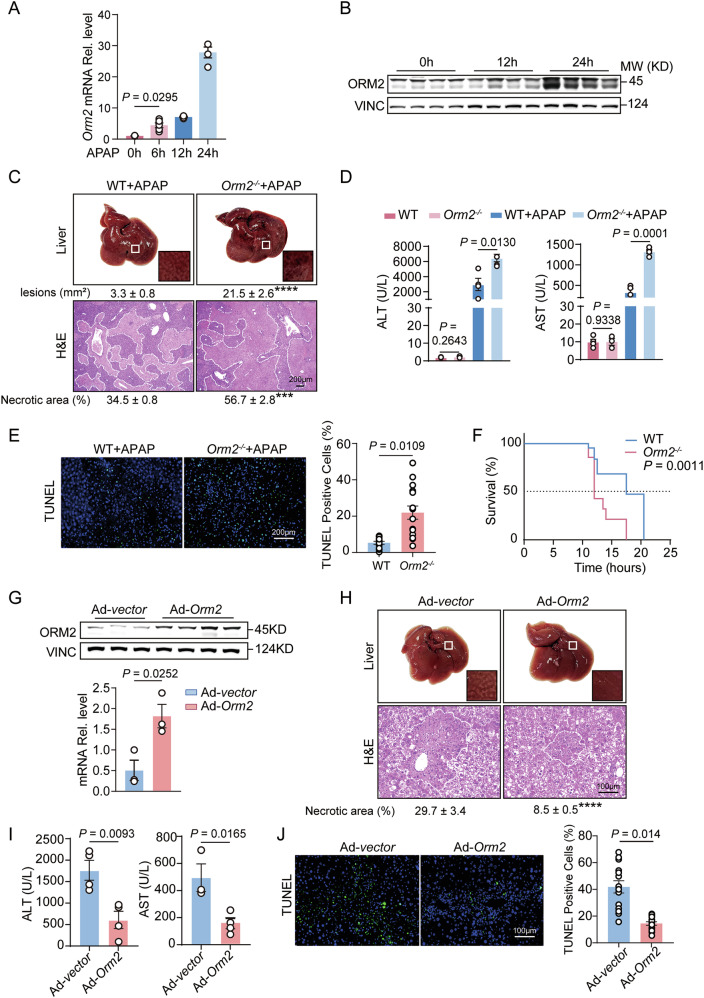
Loss of *Orm2* in hepatocytes exacerbates chemical-induced acute liver injury in mice. *Orm2* expression in livers of wild-type (WT) mice at different times following 300 mg/kg APAP treatment, measured by qPCR (**A**) (*n* = 3 (0 h), *n* = 5 (6 h), *n* = 4 (12 h and 24 h)) and western blot (**B**) (*n* = 4 per group). Vinculin (VINC) was used as the loading control. **C** Representative gross liver and H&E images from WT and *Orm2*^*-/-*^ mice at 24 h post-APAP. Scale bar, 200 μm. Necrotic areas circled in white lines, with quantification of necrosis and areas of patchy hemorrhages shown below (*n* = 4 per group). **D** Serum levels of ALT and AST in WT and *Orm2*^*-/-*^ mice at 24 h post-APAP. **E** Apoptosis measured by TUNEL staining from WT and *Orm2*^*-/-*^ mice at 24 h post-APAP. Scale bar, 200 μm. Quantification is on the right. **F** Survival curves of WT and *Orm2*^*-/-*^ mice treated with a lethal dose of APAP (750 mg/kg) (*n* = 10 per group). Eight-week-old WT male mice were injected retro-orbitally with Ad-*ORM2* or Ad-*vector* virus. After two weeks, WT and *Orm2*^*-/-*^ mice received 300 mg/kg APAP intraperitoneally. 24 h later, ORM2 protein and mRNA levels in liver tissue (**G**), liver morphology (**H**), H&E (**H**), serum ALT and AST levels (**I**), as well as TUNEL staining (**J**), were assessed (*n* = 5 per group). Scale bars, 100 μm. All data are presented as mean ± SEM. In (**A**), data were analyzed by one-way ANOVA with Dunnett’s test. In (**F**), the survival curve was compared using the log-rank (Mantel–Cox) test, and the remaining data were analyzed by unpaired two-tailed Student’s t test. Exact *p* values are provided in the figure, ***p < 0.001, ****p < 0.0001. “n” refers to biological replicates. All experiments were performed in triplicates.

To identify hepatoprotective factors during liver injury, we analyzed liver RNA-seq data from APAP-treated mice. Both published and our own RNA-seq results revealed that a series of acute response proteins, including *Orm1*, *Orm2*, and *Saa1/2*, were rapidly upregulated in the early stages of injury, suggesting their potential defensive role (Fig. S1A) [41]. Interestingly, while both *Orm*s showed increased expression during the acute phase response, *Orm1* maintained constitutively high basal levels (30-fold greater than ORM2). In contrast, *Orm2* exhibited dramatic injury-specific upregulation (8-10 fold induction, p < 0.001) during acute liver injury compared to the basal condition, suggesting a more context-specific response (Fig. S1B). We then confirmed that *Orm2* mRNA and protein expression rose rapidly throughout the time after APAP treatment (Fig. 1A–B). *Orm2* is predominantly expressed in hepatocytes, we generated *Orm2* knockout (*Orm2* KO, *Orm2*^*−/−*^) mice [37] to test the functional influence of ORM2 in the liver injury. The *Orm2* KO mice were healthy at baseline, with liver function indicators (AST/ALT) comparable to wild-type (WT) mice [37]. Twenty-four hours after APAP treatment, quantification revealed significantly more visible patchy hemorrhages on both the surface and interior of the liver, with some areas appearing pale in *Orm2* KO mice (21.5 ± 2.6 lesions/mm²) compared to the controls (3.3 ± 0.8 lesions/mm²; *p* < 0.001, *n* = 4/group) (Fig. 1C). Hematoxylin and eosin (H&E) staining revealed more extensive hepatocyte necrosis in the liver tissue of *Orm2* KO mice (Fig. 1C). Serum markers of liver injury, AST and ALT, were significantly elevated, and TUNEL staining showed increased cell death in *Orm2* KO mice (Fig. 1D–E). In a high-dose APAP model simulating acute liver failure, *Orm2* knockout dramatically shortened the median survival of mice than the WT controls (Fig. 1F). Similarly, in a CCl_4_-induced mouse model, loss of *Orm2* further exacerbated acute liver injury compared to WT (Fig. S1C–E). We then overexpressed *Orm2* in mouse livers using adenoviral delivery (Fig. 1G). Compared to the empty vector control, *Orm2*-overexpressing mice showed notably reduced necrotic areas in liver tissue, improved liver function, and fewer TUNEL-positive cells (Fig. 1H–J). Furthermore, using hydrodynamic gene transfection method, we overexpressed *Orm1* specifically in the liver with an efficiency comparable to *Orm2*. However, unlike *Orm2*, *Orm1* failed to confer protection against APAP-induced hepatotoxicity (Fig. S1F–S1G). Collectively, these results demonstrate that chemical-induced acute liver injury upregulates *Orm2* expression in WT mice, whereas *Orm2* deficiency aggravates liver injury. These findings suggest that *Orm2* mediates a potential defensive response against APAP-induced acute hepatotoxicity, alleviating rapid homeostasis dysregulation.

### Hepatocyte *Orm2* loss promotes ferrous ion accumulation and toxicity in APAP-induced liver injury

APAP primarily undergoes phase II metabolism to form non-toxic conjugates. However, approximately 10% is bioactivated by cytochrome P450 enzymes (e.g., CYP2E1) to generate the highly reactive metabolite N-acetyl-p-benzoquinone imine (NAPQI). NAPQI forms covalent adducts with proteins or DNA, disrupts mitochondrial function, and induces reactive oxygen species (ROS) [42, 43]. Next, we examined the biotransformation process of APAP in the liver. The results showed that both the mRNA and protein levels of CYP2E1 and the levels of NAPQI (metabolite in the early phase) were similar between WT and *Orm2* KO mice (Fig. S2A–S2C). We then examined and found that there were no significant differences in the enzyme activity of Glutathione Reductase (GR) and Glutathione S-transferase (GST) (metabolic activation in the early phase), the mRNA expression of phase II metabolic enzymes, and the mRNA expression of antioxidant enzymes between the two groups of mice, before and after APAP treatment (Fig. S2D–S2F). These findings indicate that *Orm2* deficiency does not affect the intracellular biotransformation or the expression of antioxidant enzymes in response to APAP.

**Fig. 2:**
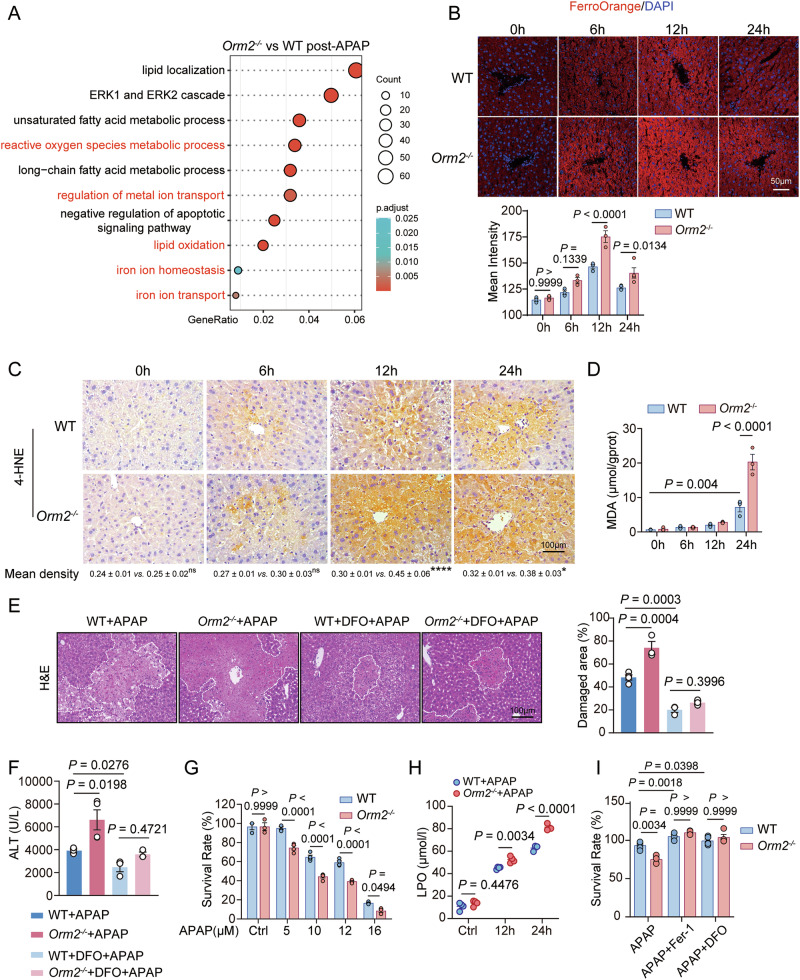
*Orm2* deficiency in hepatocytes aggravates ferroptosis in acute liver injury. **A** Differentially enriched gene pathways from RNA-seq analysis of liver tissues in *Orm2*^*-/-*^ versus WT mice 24 h after APAP treatment (*n* = 3 per group). **B** WT and *Orm2*^*-/-*^ mice were injected intraperitoneally with APAP (300 mg/kg). Liver samples were collected at the indicated time points for frozen section preparation. Sections were stained with FerroOrange to detect ferrous iron, with DAPI counterstaining. Mean FerroOrange fluorescence intensity is quantified below (*n* = 3 per group, except 24 h: *n* = 4). Scale bars, 50 μm. **C** WT and *Orm2*^*-/-*^ mice were received APAP (300 mg/kg). Liver samples were collected at the indicated time points for frozen section preparation. Representative immunohistochemical staining for 4-HNE in liver sections, with mean intensity is quantified below (*n* = 3 per group). Scale bars, 100 μm. **D** Measurement of malondialdehyde (MDA) levels in liver tissue from (**C**) (*n* = 3 per group). **E** WT and *Orm2*^*-/-*^ mice were pre-treated with 200 mg/kg DFO or vehicle 2 h before APAP injection, with samples collected 24 h post-APAP. Representative H&E images of liver sections from mice, white dashed lines indicating cell death areas (*n* = 4 per group). Scale bar, 100 μm. Damage areas were quantified on the right. **F** Serum ALT levels from mice in (**E**) were measured. **G** Primary hepatocytes isolated from WT and *Orm2*^*-/-*^ mice were treated with indicated concentrations of APAP and cell viability was measured using Cell Counting Kit 8 (CCK8). **H** Primary hepatocytes from WT and *Orm2*^*-/-*^ mice were treated with 5 μM APAP for the indicated times. LPO levels were measured. **I** Primary hepatocytes isolated from WT and *Orm2*^*-/-*^ mice were treated with 5 μM APAP in the presence of Fer-1 (2 μM) or DFO (50 μM), and cell viability was measured using CCK8. All data in this figure are represented as mean ± SEM. Data were analyzed by two-way ANOVA followed by Bonferroni’s multiple comparisons test for comparisons between genotypes, and by one-way ANOVA with Dunnett’s test or unpaired t-test for comparisons between two or multiple experimental groups within the same genotype, respectively. Exact *p* values are provided in the figure, *p < 0.05, ****p < 0.0001, ns (no significance). All experiments were performed in triplicates.

Next, we asked if *Orm2* influences hepatocyte death following injury. Western blot and immunohistochemistry (IHC) results showed no differences in cleaved Caspase-3 levels with *Orm2* loss (Fig. S2G-S2H). The phospho-mixed lineage kinase domain-like pseudokinase (phospho-MLKL), a necroptosis marker, also indicated that *Orm2* is not involved in cell programmed necroptosis (Fig. S2I). We then analyzed RNA-seq results on liver tissues from WT and *Orm2* KO mice 24 h after APAP treatment. Differential gene analysis revealed significant enrichment of multiple pathways related ROS generation and iron metabolism in *Orm2* KO mice compared to WT mice (Fig. 2A), indicating that *Orm2* might influence iron metabolism in injured livers. By using FerroOrange immunofluorescence staining on frozen liver sections, we assessed free ferrous ions, which are toxic ions that promote peroxidation. Additionally, we examined lipid peroxidation using IHC staining for 4-Hydroxynonenal (4-HNE) and measured malondialdehyde (MDA) levels in liver homogenates using an MDA assay kit. The results showed that, over the course of APAP induction, the levels of free ferrous ions and lipid peroxidation progressively increased in the liver sections of *Orm2* KO mice compared to control mice (Fig. 2B–D and Fig. S2J). In addition, GPX4 protein levels decreased, indicating GSH exhaustion (Fig. S2K). Furthermore, treatment with the iron chelator deferoxamine mesylate (DFO) significantly alleviated the injury phenotype in both groups of mice (Fig. 2E–F and Fig. S2L). These results indicate that the exacerbated liver injury induced by APAP in *Orm2* KO mice is not mediated by upregulation of apoptotic and necroptotic pathways but by promoting ferrous overload, leading to ferroptosis.

To further clarify whether increased sensitivity to APAP-induced injury in *Orm2*-deficient liver tissues is due to the loss of *Orm2* in hepatocytes, we isolated primary hepatocytes from WT and *Orm2* KO mice and assessed their sensitivity to APAP. The hepatocytes were treated with APAP at varying concentrations and exposure times. Results demonstrated significantly reduced viability, while elevated lipid peroxidation levels in *Orm2* KO hepatocytes, compared to WT cells (Fig. 2G–H and Fig. S2M). This phenotype was reversed with ferrostatin-1 (Fer-1) or DFO treatment (Fig. 2I). These results are consistent with the in vivo findings and indicate that hepatocytes lacking *Orm2* are more susceptible to APAP-induced cell death.

### Loss of ORM2 increases cellular sensitivity to ferroptosis inducers

**Fig. 3:**
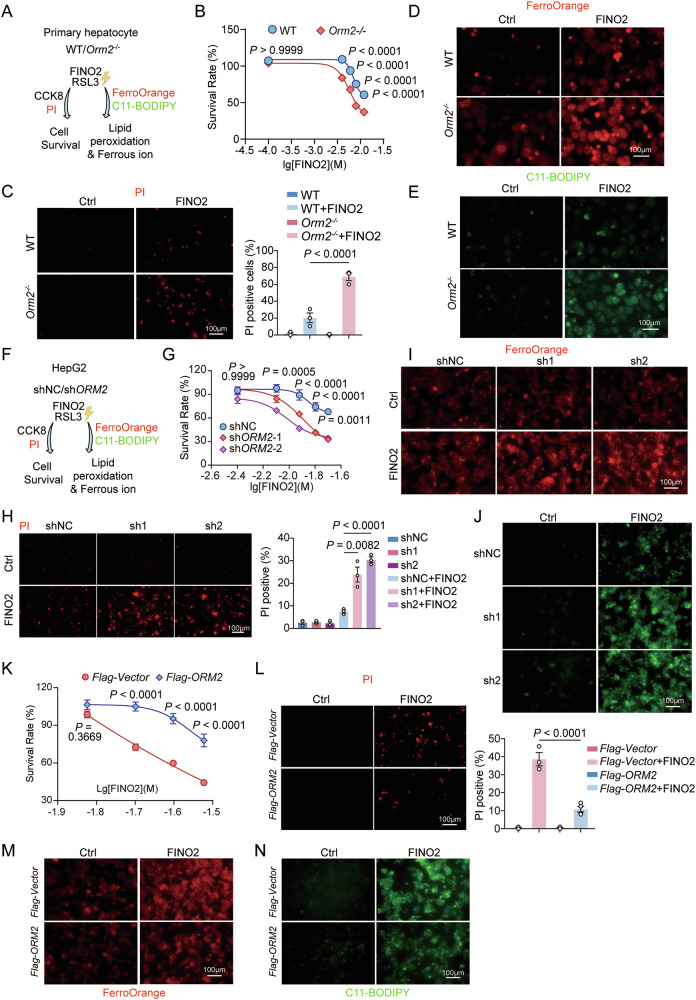
Loss of *ORM2* increases cellular sensitivity to ferroptosis inducers. **A** The schema illustrates primary hepatocytes isolated from WT and *Orm2*^*-/-*^ mice, then treated with FINO2 or RSL3, with cell survival, cell death, ferrous iron, and lipid peroxidation levels measured. **B** WT and *Orm2*^*-/-*^ primary hepatocytes were treated with indicated doses of FINO2 for 12 h, cell viability was measured using CCK8. **C** WT and *Orm2*^*-/-*^ primary hepatocytes were treated with 8 μM FINO2 for 12 h, and propidium iodide (PI) staining to assess cell death. Quantification on the right. Scale bar, 100 μm. **D**–**E** WT and *Orm2*^-/-^ primary hepatocytes were treated with 8 μM FINO2 for 3 h, Fe^2+^ levels and lipid peroxidation were visualized using FerroOrange and C11-BODIPY staining respectively. Scale bar, 100 µm. **F** The schema illustrates HepG2 cells with *ORM2* knockdown, cells were treated with FINO2 or RSL3, with cell survival, cell death, ferrous iron, and lipid peroxidation levels measured. **G** HepG2 cells with *ORM2* knockdown were treated with FINO2 at the indicated concentrations for 12 h, and cell viability was assessed using CCK8. **H** HepG2 cells with *ORM2* knockdown were treated with 8 μM FINO2 for 12 h, PI staining was measured. Quantification on the right. Scale bar, 100 μm. **I**–**J** HepG2 cells with *ORM2* knockdown were treated with 8 μM FINO2 for 3 h, Fe^2+^ levels and lipid peroxidation were visualized using FerroOrange and C11-BODIPY staining respectively. Scale bar, 100 μm. **K** *Flag-ORM2* was overexpressed into HepG2 cells, cells were treated with FINO2 at indicated doses for 12 h. Cell viability was assessed using CCK8. **L** HepG2 cells with *ORM2* overexpressing were treated with 8 μM FINO2 for 12 h, PI staining was measured. Quantification on the right. Scale bar, 100 μm. **M**–**N** HepG2 cells with *ORM2* overexpressing were treated with 8 μM FINO2 for 3 h, Fe^2+^ levels and lipid peroxidation were visualized using FerroOrange and C11-BODIPY staining, respectively. Scale bar, 100 μm. All data in this figure are represented as mean ± SEM. Data were analyzed by two-way ANOVA followed by Bonferroni’s multiple comparisons test for comparisons between genotypes. Exact *p* values are provided in the figure. All experiments were performed in triplicates.

To test if ORM2 influence susceptibility to ferroptosis, we treated primary hepatocytes from WT and *Orm2* KO mice with various concentrations of ferroptosis inducers FINO2 or RSL3 (Fig. 3A). The results showed that the survival rate of cells in the *Orm2* KO group was consistently lower than that of control hepatocytes. This was accompanied by higher levels ferrous ion, amplified cell death signaling, and exacerbated lipid peroxidation (Fig. 3B–E and Fig. S3A–S3B). We then mimicked the *Orm2* deficiency using lentiviral-mediated shRNA to knock down *ORM2* in the human liver cancer cell line HepG2 (Fig. 3F and Fig. S3C). Both independent shRNAs markedly reduced ORM2 mRNA expression to extremely low levels (less than 0.5% of the control), demonstrating the high efficacy of the knockdown constructs (Fig. S3C). Consistently, after treatment with different concentrations of FINO2 or RSL3, *ORM2* knockdown resulted in significantly increased cellular sensitivity to ferroptosis, showing elevated ferrous iron accumulation, increased cell death and exacerbated lipid peroxidation (Fig. 3G–J and Fig. S3D–G). Furthermore, we treated HepG2 cells with cisplatin to induce apoptosis and found that *ORM2* knockdown did not affect the cellular sensitivity to apoptosis (Fig. S3H). Additionally, we transfected the *ORM2* plasmid into HepG2 or the mouse immortalized hepatocyte cell line AML12, creating stable *ORM2* overexpression cell lines. *ORM2* overexpression impaired FINO2-induced cell death, accompanied by decreased ferrous iron levels and lipid peroxidation (Fig. 3K–N and Fig. S3I). Similarly, AML12 cells overexpressing *ORM2* showed reduced susceptibility to erastin-induced cell death (Fig. S3J–S3K). Collectively, these results demonstrate that ORM2 exerts a protective effect in the context of ferroptosis.

### ORM2 suppresses ferritin degradation by disrupting the NCOA4-TAX1BP1- mediated ferritinophagy

**Fig. 4:**
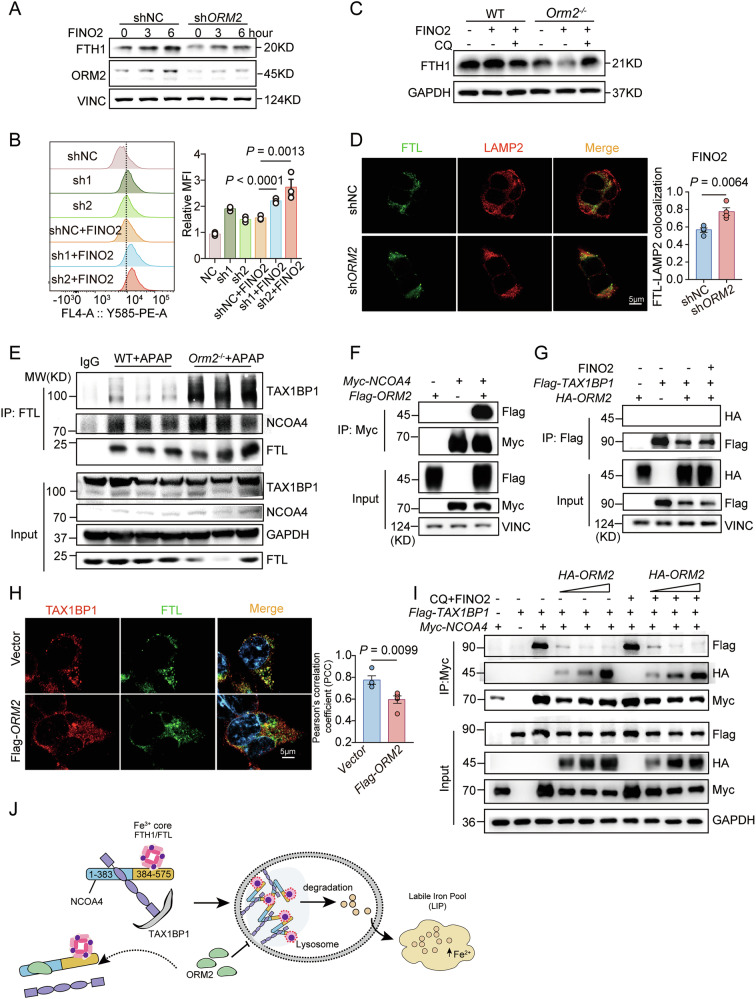
*ORM2* suppresses ferritinophagy by disrupting the NCOA4-TAX1BP1 interaction. **A** HepG2 cells with shNC or sh*ORM2* knockdown were treated with 8 μM FINO2 for the indicated time points, western blot to detect FTH1 and ORM2 protein expression. **B** HepG2 cells with shNC or sh*ORM2* knockdown were treated with 8 μM FINO2 for 6 h. The intracellular ferrous ion (Fe^2+^) levels were stained with FerroOrange (0.25 µmol/L) and analyzed by flow cytometry. **C** Primary hepatocytes were isolated from WT and *Orm2*^*-/-*^ mice, and cells were treated with 8 μM FINO2 and 10 μM Chloroquine (CQ). FTH1 protein expression was measured by western blot. **D** HepG2 cells with *ORM2* knockdown or control were treated with 8 μM FINO2. Representative immunofluorescence images display ferritin (FTL) and lysosomes (LAMP2 positive foci) localization. Quantification of co-localization was analyzed using Image J (right panel). Scale bars, 5 μm. **E** WT and *Orm2*^*-/-*^ mice were injected with APAP. 24 h post-APAP, liver tissues were collected and lysed for FTL co-immunoprecipitation (co-IP), and TAX1BP1, NCOA4, FTL, and GAPDH antibodies were used for IB. **F** HEK293T cells were co-transfected with *Flag-ORM2* and *Myc-NCOA4* plasmids. Cells were treated with 8 μM FINO2 or vehicle for 3 h, followed by co-IP using Myc beads. Flag-ORM2 levels in the co-IP complexes were analyzed. **G** HEK293T cells were co-transfected with *Flag-TAX1BP1* and *HA-ORM2*. Cells were treated with 8 μM FINO2 or vehicle for 3 h, followed by co-IP using Flag beads. HA-ORM2 protein were measured. **H** HepG2 cells were overexpressed with *Flag-ORM2* plasmid. Cells were treated with 4 μM RSL3. Representative immunofluorescence images of TAX1BP1 and FTL were detected, and the Pearson correlation for co-localization was quantified using ImageJ. Scale bars, 5 μm. **I** HEK293T cells were co-transfected with *Flag-TAX1BP1*, *Myc-NCOA4*, and increasing amounts of *HA-ORM2* plasmids. Cells were then treated with 8 μM FINO2 plus 10 μM chloroquine (CQ) or DMSO vehicle. Co-IP were performed using Myc beads, and Flag, HA, Myc, and GAPDH antibodies were used for IB. **J** A model illustrates that ORM2 inhibits ferritin localization to lysosomes during ferroptosis by modulating NCOA4-TAX1BP1 interactions, reducing intracellular ferrous ions. All data in this figure are represented as mean ± SEM. In (**B**), data were analyzed by two-way ANOVA followed by Bonferroni’s multiple comparisons test; in (**D**) and (**H**), by unpaired *t*-test. Exact *p* values are provided in the figure. All experiments were performed in triplicates.

The RNA-seq results indicated alterations in several iron ion transport and homeostasis pathways in ORM2-deficient livers (Fig. 2A), we then examined whether ORM2 influence iron transport and metabolism under stress conditions. Comparative analysis revealed that, upon FINO2 treatment, *ORM2*-knockdown cells exhibited significant reductions in Transferrin receptor protein 1 (TFR1), and ferritin heavy chain (FTH1) protein levels; but increased Solute Carrier Family 7 Member 11 (SLC7A11) expression (Fig. S4A and Fig. 4A). However, only the decrease in FTH1 could explain the enhanced ferroptosis susceptibility observed in *ORM2*-knockdown cells and mouse injury models. Thus, this regulatory pattern strongly suggests that ORM2 protects against ferroptosis, at least in part, by specifically stabilizing FTH1 protein levels. Consistently, *Orm2* knockout significantly reduced ferritin protein levels in the liver after APAP treatment (Fig. S4B–S4D). Since the downregulation of ferritin proteins results in the release of intracellular free ferrous ions into the cellular labile iron pool, we measured ferrous ion levels using flow cytometry. The results showed that knocking down *ORM2* in HepG2 cells significantly increased intracellular ferrous ion levels following FINO2 treatment (Fig. 4B). Altogether, our findings suggest that loss of *Orm2* reduces ferritin content, promotes ferrous ion release, and increases the cellular labile iron pool, leading to heightened cellular sensitivity to ferroptosis inducers.

Next, we assessed the underlying molecular mechanism by which ORM2 influences ferritin levels. RNA-seq and qPCR analysis showed no reduction in ferritin gene expression in APAP-treated *Orm2*^*-/-*^ mice compared with WT controls (Fig. S4E). Next, we examined ferritin protein degradation. Ferritin is typically degraded through two lysosomal pathways: macroautophagy and ESCRT-dependent endosomal microautophagy [44–46]. We isolated primary hepatocytes and treated them with FINO2 in combination with the autophagy inhibitor chloroquine (CQ). The results revealed that loss of *Orm2* led to a reduction in ferritin (FTH1) protein levels compared to wildtype cells, particularly in the context of FINO2 induction, and mediated through ferritinophagy (Fig. 4C). Next, the immunofluorescence results showed that *ORM2* knockdown increased ferritin co-localization with lysosomes, as indicated by its overlay with LAMP2 (lysosomal-associated membrane protein 2)-positive foci, in HepG2 cells treated with FINO2 or RSL3 (Fig. 4D and Fig. S4F). This suggests *ORM2* loss promotes ferritinophagy within lysosomes during ferroptosis induction. As previously reported, during ferritinophagy, ferritin primarily forms the complex with the receptor NCOA4, then recognized by the adaptor TAX1BP1 and degraded in the lysosome [47]. Therefore, we examined the formation of the ferritin-NCOA4-TAX1BP1 complex. Co-immunoprecipitation (co-IP) assay demonstrated that the interaction between ferritin and TAX1BP1 was robustly enhanced in *Orm2* KO livers 24 h after APAP treatment, while the interaction between ferritin and NCOA4 remained unchanged (Fig. 4E). Next, co-IP assays demonstrated that ORM2 interacts with NCOA4 (Fig. 4F). In contrast, ORM2 did not interact with TAX1BP1, either with or without FINO2-induced stress (Fig. 4G). Furthermore, overexpression of *ORM2* reduced the co-localization of ferritin and TAX1BP1 (Fig. 4H). In consistent, overexpression of *ORM2* weakened the interaction between NCOA4 and TAX1BP1 (Fig. 4I). These results suggests that ORM2 may competitively bind to NCOA4, thereby inhibiting TAX1BP1’s interaction with NCOA4/ferritin.

To further elucidate the molecular basis of the ORM2-NCOA4 interaction, we performed systematic binding studies using NCOA4 truncations based on its domain architecture (Fig. S5A) [48, 49]. Our results demonstrate that both ORM2 and TAX1BP1 bind to the N-terminal segment (aa 1-383) of NCOA4, while a shorter N-terminal truncation (aa 1-238) failed to maintain stable binding with either protein (Fig. S5B–S5C). Interestingly, the C-terminal truncation (aa 238-575) retained binding capability with ORM2 but not TAX1BP1 (Figs. S5B–S5C), suggesting that ORM2 primarily interacts with residues 238-383 of NCOA4 whereas TAX1BP1 requires structural elements spanning residue 238 for binding. Furthermore, ORM2 overexpression disrupted the interaction between the N383 truncation of NCOA4 and TAX1BP1, mirroring its effect on the full-length protein (Fig. S5D). Taken together, these studies provide evidence for a competitive interaction of ORM2 and TAX1BP1 with the N-terminal sequences (across aa238) of NCOA4.

Next, we examined if there is the potential interaction between ORM2 and other key ferroptosis regulators. The results revealed that ORM2’s interaction is highly specific: it binds strongly and consistently to NCOA4, exhibits a weaker, signal-modulated association with SLC7A11 (which is attenuated upon FINO2 treatment), and shows no interaction with other regulators, including FSP1, GPX4, or TAX1BP1 (Fig. S5E). These findings significantly demonstrate that ORM2’s protection is not mediated through a broad, non-specific engagement with multiple ferroptosis pathways. Instead, it operates through a precise and selective mechanism, primarily targeting the NCOA4-mediated ferritinophagy axis to limit ferrous iron overload. Moreover, *ORM2* knockdown decreased cell viability; however, additional knockdown of TAX1BP1 restored cell viability under ferroptosis conditions (Fig. S5F). In summary, ORM2 competitively binds to a region within the N-terminal segment (aa 1-383) of NCOA4, thereby sequestering NCOA4 from TAX1BP1. This interaction blocks the recruitment of the ferritin-NCOA4 complex by TAX1BP1, ultimately preventing the lysosomal targeting and autophagic degradation of ferritin under stress conditions (Fig. 4J).

### The application of recombinant ORM2 protein significantly reduces acute liver injury

ORM2 is an acute-phase protein that is rapidly synthesized and secreted from the liver in response to tissue injuries. We observed a notable increase in serum ORM2 levels following APAP treatment in mice (Fig. S6A). Thus, we investigated whether paracrine ORM2 could be taken up by neighboring hepatocytes and protect the local liver tissue under stress. To test this, we first treated primary hepatocytes with RSL3 while adding recombinant ORM2 protein on media. The results demonstrated that ORM2 treatment protected hepatocytes from RSL3 induced-ferroptosis and oxidative stress (Fig. S6B–E). Given that ORM2 is a secreted protein, we then also investigated whether cellular uptake is required for its cytoprotective function. To test this, we treated cells with recombinant ORM2 protein in the presence or absence of amiloride, a macropinocytosis inhibitor, to block potential endocytic entry. We found that it could reverse the protective effect of the recombinant ORM2 protein (Fig. S6B–E). Consistent protective findings were observed in HepG2 cell lines, where ORM2 recombinant protein treatment led to significant improved cell survival, reduced ROS levels, and decreased ferrous ion release after RSL3 (Fig. S6F–H). These results suggest that ORM2 proteins can be internalized by cells via endocytosis, thereby regulating iron homeostasis and exerting protective effects against ferroptosis.

**Fig. 5:**
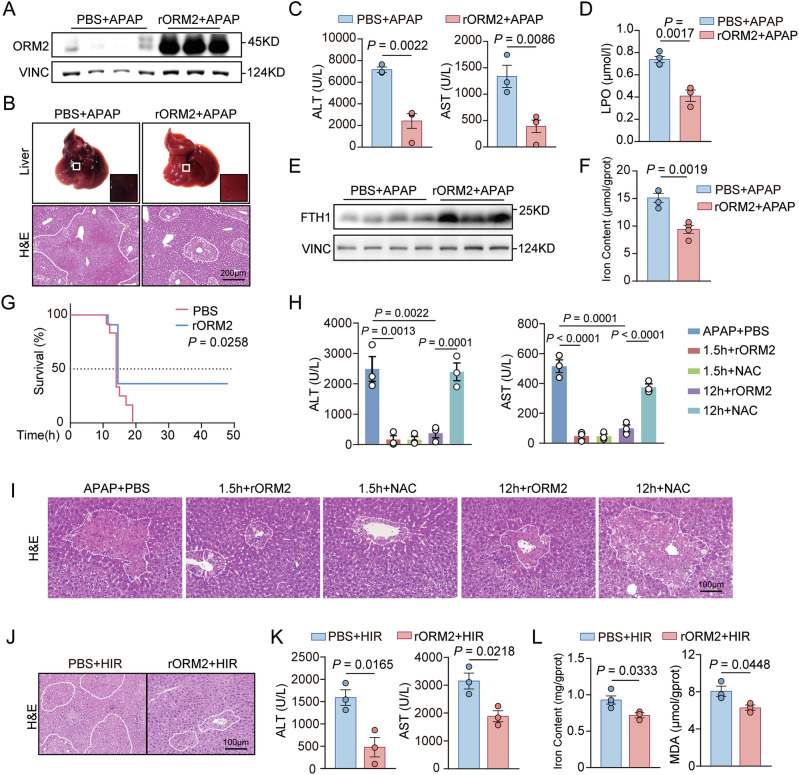
Infusion of recombinant ORM2 protein significantly alleviates acute liver injury. **A** WT mice were pre-injected intraperitoneally with recombinant ORM2 protein (rORM2, 7 mg/kg) or PBS for 2 h, followed by 300 mg/kg APAP. After 24 h, liver samples were collected for ORM2 expression analysis by western blot (*n* = 4 and 3). **B** Representative liver morphology and H&E images of livers from mice in (**A**). Scale bar, 200 μm. **C**–**F** Serum ALT, AST, LPO, liver FTH1 protein and liver iron levels were measured from mice in (**A**), (*n* = 4-3 per group). **G** WT mice received 7 mg/kg rORM2 or PBS 2 h before 750 mg/kg APAP, and survival curves were plotted, (*n* = 12 per group). **H** WT mice were injected intraperitoneally with APAP (250 mg/kg) to induce liver injury. At 1.5- and 12-h post-APAP injection, mice received equal volumes of PBS, rORM2 (20 mg/kg), or NAC (300 mg/kg) as indicated. Serum samples were collected at 24 h after APAP, serum ALT and AST were measured (*n* = 3 per group). **I** Representative H&E staining of liver sections from the indicated groups in (**H**). Scale bar, 100 μm. **J** WT mice received 7 mg/kg rORM2 2 h before performing liver ischemia-reperfusion (HIR). 12 h post-HIR, livers were collected for H&E, the white dashed lines indicated the damaged area (*n* = 3 per group). Scale bar, 100 μm. **K**–**L** Serum ALT, AST, liver iron content, and liver MDA from mice in (**J**) were measured, (*n* = 3 per group). All data in this figure are represented as mean ± SEM. In (**G**), the survival curve was compared using the log-rank (Mantel-Cox) test; In (**H**) data were analyzed by two-way ANOVA followed by Bonferroni’s multiple comparisons test for comparisons between rORM2 and NAC groups, and by one-way ANOVA with Dunnett’s test for comparisons between APAP + PBS and rORM2+APAP groups; the remaining data were analyzed by unpaired two-tailed Student’s *t* test. Exact *p* values are provided in the figure. “n” refers to biological replicates. All experiments were performed in triplicates.

Next, we administered recombinant ORM2 protein (long-acting recombinant ORM2-Fc) to mice 2 h prior to APAP treatment (Fig. 5A). The Fc portion of the fusion protein engages the neonatal Fc receptor (FcRn), a well-established strategy for therapeutic proteins that mediates cellular recycling and reduces systemic clearance, thereby prolonging its in vivo presence [50, 51]. The results demonstrated that recombinant ORM2 protein significantly alleviated APAP-induced acute liver injury, reduced lipid peroxide levels, increased ferritin level in livers and reduced iron accumulation (Fig. 5B–F). Notably, in the APAP-induced liver failure model, recombinant ORM2 protein intervention significantly improved the survival of mice than the vehicle control group (Fig. 5G). N-acetylcysteine (NAC) is a potent protective agent against APAP-induced liver injury on clinic. However, its therapeutic window is extremely narrow. Studies have reported that NAC is effective only when administered within 1.5 h after liver injury, significantly limiting its clinical application in treating such conditions. Next, we compared the therapeutic effects of ORM2 protein intervention with those of NAC treatment. The results demonstrated that both NAC and ORM2 protein application, administered 1.5 h after APAP induction, significantly reduced liver injury. However, ORM2 treatment exhibited notably superior therapeutic efficacy compared to NAC when the intervention was delayed to 12 h post-APAP induction (Fig. 5H–I). In summary, these results suggest that circulating ORM2 protein plays a critical role in protecting the injured liver, reducing liver injury and lowering the risk of liver failure. Furthermore, it also indicates that additional exogenous ORM2 treatment is an effective strategy to inhibit APAP-induced liver injury.

Additionally, we evaluated the function of recombinant ORM2 protein in a liver ischemia-reperfusion model, a well-established system for study ferroptosis pathology. Pre-treatment with recombinant ORM2 resulted in significantly better liver function, reduced iron accumulation, and decreased lipid peroxidation (Fig. 5J–L). These results further highlight the importance of ORM2 as a key factor in mitigating ferroptosis-driven damage and maintaining liver function under stress conditions.

### Administration of recombinant ORM2 protein protects multiple organs from ischemia-reperfusion injury by inhibiting ferroptosis

**Fig. 6:**
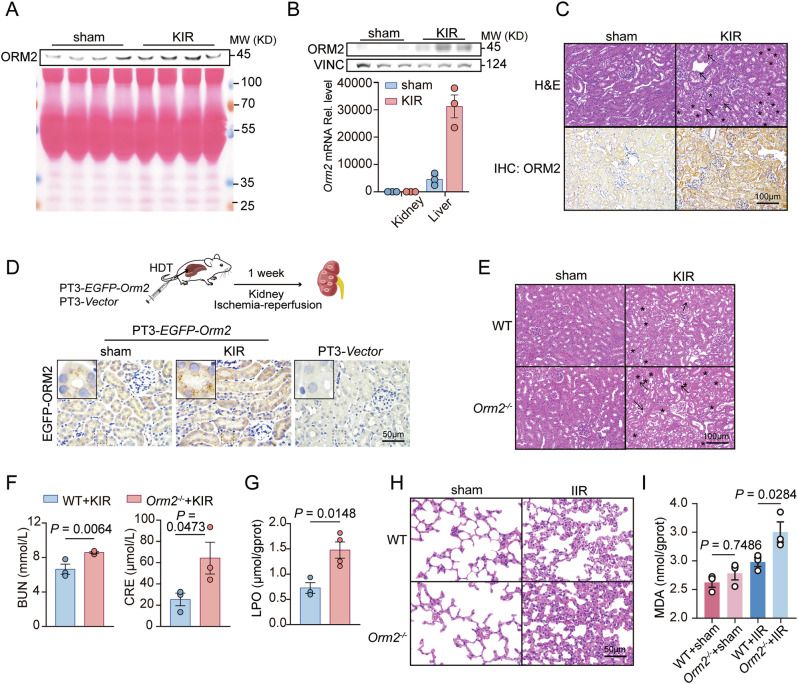
Loss of ORM2 in the liver exacerbates ischemia-reperfusion injury and increases tissue ferroptosis in distant organs. **A** Wild-type mice underwent sham or renal ischemia-reperfusion (KIR) surgery. Serum samples were collected at 12 h. ORM2 protein level of the serum was quantified relative to total protein using Ponceau S staining and western blot (*n* = 4 per group). **B** Kidney ORM2 protein levels; liver and kidney *Orm2* mRNA from (**A**) were measured by western blot and qPCR. **C** Wild-type mice underwent sham or renal ischemia-reperfusion (KIR) surgery, kidney samples were collected for H&E and ORM2 staining. H&E staining shows swelling (*) and nuclear condensation (→) of renal tubular epithelial cells (*n* = 3 per group). Scale bars, 100 μm. **D** Sleeping beauty transposon PT3-Vector or PT3-*Orm2-EGFP* and *SB100* transposase were delivered via hydrodynamic tail vein injection (HDT) in mice. One week later, mice underwent either sham or KIR surgery. EGFP-ORM2 expression in kidney sections was assessed by immunohistochemistry (*n* = 3 per group). Scale bars, 50 μm. **E** WT and *Orm2*^*-/-*^ mice underwent KIR surgery, kidney samples were collected 12 h post-surgery. H&E staining shows swelling (*) and nuclear condensation (→) of renal tubular epithelial cells (*n* = 3 (WT mice), *n* = 4 (*Orm2* ^*-/-*^ mice)). Scale bars, 100 μm. **F** Serum creatinine and blood urea nitrogen (BUN) levels of mice in (**E**) were measured 12 h post-KIR. **G** Lipid peroxidation (LPO) in kidney tissues of mice in (**E**) (*n* = 3 per group). **H** WT and *Orm2*^*-/-*^ mice underwent intestinal ischemia-reperfusion (IIR) surgery, lung samples were collected 3 h post-surgery. Images show H&E staining of lung sections (*n* = 3 per group). Scale bars, 50 μm. **I** Malondialdehyde (MDA) levels of lung samples from mice in (**H**) (*n* = 3 per group). All data in this figure are represented as mean ± SEM. In (**I**), data were analyzed by two-way ANOVA followed by Bonferroni’s multiple comparisons test; the remaining data were analyzed by unpaired two-tailed Student’s *t* test. Exact *p* values are provided in the figure. “n” refers to biological replicates. All experiments were performed in triplicates.

Previous study suggests that ORM protects against kidney ischemia-reperfusion injury [40]. Therefore, we then investigate whether the liver respond to distant organ injury by secreting ORM2 as a defense mechanism against ferrous toxicity. Indeed, in a renal ischemia-reperfusion injury model, we observed significant increases in serum ORM2 protein levels, liver expression, and the amount of ORM2 entering the kidney (Fig. 6A–C). Using hydrodynamic tail vein injection (HDT) method, we overexpressed *ORM2-EGFP* fusion plasmid in mouse liver. After renal ischemia-reperfusion, EGFP staining revealed a marked increase in ORM2 protein in kidney tissues, indicating a significant secretion of ORM2 by the liver in response to renal injury and liver-secreted ORM2 is endocytosed by renal epithelial cells (Fig. 6D).

Next, we performed renal ischemia-reperfusion modeling on *Orm2* KO mice. The results showed that *Orm2* deficiency significantly aggravated kidney injury, as indicated by deteriorated tissue histology, abnormal indicators of kidney function, and elevated lipid peroxidation levels (Fig. 6E–G). Next, we detected another classic ferroptosis model by constructing an acute lung injury model caused by intestinal ischemia-reperfusion. The results showed that loss of *Orm2* significantly enhanced thickening of alveolar septa and increased inflammatory cell infiltration, as well as lipid peroxidation levels in lung tissue (Fig. 6H–I). These results suggest that liver cross-talked with other damaged organs during ischemia-reperfusion injury via the secretion of ORM2, which demonstrates protective effects in multiple tissues.

**Fig. 7:**
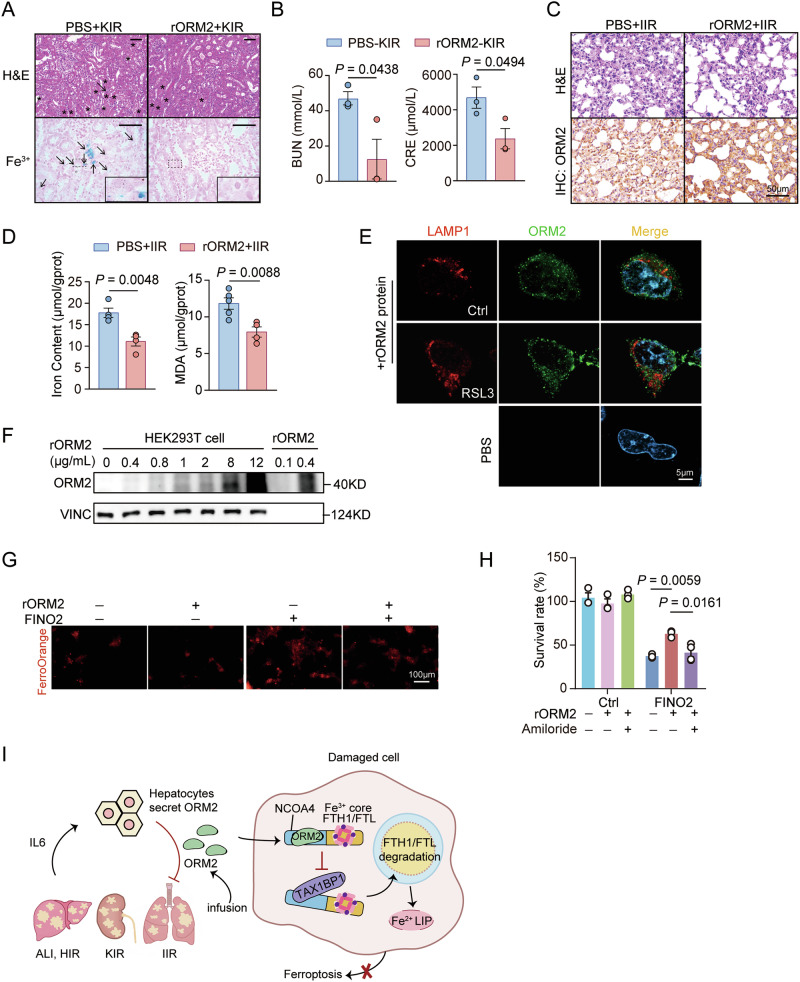
Infusion of recombinant ORM2 protein protects multiple organs from ischemia-reperfusion injury by inhibiting ferroptosis. **A** Wild-type mice received 7 mg/kg recombinant mouse ORM2-Fc protein (rORM2) 2 h before KIR. Kidney sections stained with H&E and Prussian blue. Scale bars, 100 μm. **B** Urine creatinine and blood urea nitrogen (BUN) levels at 12 h from mouse urine samples in (**A**) (*n* = 3 per group). **C** Wild-type mice received 7 mg/kg rORM2 2 h before intestinal I/R (IIR), lung samples were collected at 3 h post-surgery and stained for H&E and ORM2 (*n* = 4 per group). Scale bars, 50 μm. **D** Iron and MDA levels of lung samples from (**C**) are presented (*n* = 4 per group). **E** HEK293T cells were incubated with 1 μg/mL rORM2 protein with or without 1 μM RSL3 treatment for 6 h. Images show rORM2 and LAMP1 localization. Scale bars, 5 μm. **F** Western blot analysis of rORM2 levels in HEK293T cells after an 8-h incubation with the indicated concentrations of ORM2 recombinant protein in the media. **G** HEK293T cells were pretreated with 1 μg/mL rORM2 proteins for 2 h, followed by 8 μM FINO2 for 6 h. FerroOrange staining was used to assess ferrous ion levels. Scale bars, 100 μm. **H** HEK293T cells were treated with Amiloride (2 μM) for 2 h, then incubated with or without 1 μg/mL rORM2 for 2 h. Cells were then exposed to 8 μM FINO2 for 12 h, and cell viability was measured using CCK8 assay. **I** A model suggests that ischemia-reperfusion injuries increase hepatic ORM2 production and secretion. Circulating or recombinant ORM2 protein then mitigates the injury by inhibiting ferroptosis. All data in this figure are represented as mean ± SEM. In (**H**), data were analyzed by two-way ANOVA followed by Bonferroni’s multiple comparisons test; the remaining data were analyzed by unpaired two-tailed Student’s *t* test. Exact *p* values are provided in the figure. “n” refers to biological replicates. All experiments were performed in triplicates.

Therefore, we explored the broad protective potential of administering ORM2 protein. We pre-treated mice with the recombinant ORM2 protein before performing renal ischemia-reperfusion injury. The results showed that mice receiving additional ORM2 exhibited less severe injury and reduced tissue iron levels (Fig. 7A–B). Similarly, in the lung injury model induced by intestinal ischemia-reperfusion, ORM2 intervention significantly mitigated the extent of lung injury, reducing both tissue iron ion and lipid peroxidation levels (Fig. 7C–D). These results confirm that recombinant ORM2 can protect multiple damaged tissues from ferrous overload and ferroptosis.

To further evaluate the anti-ferroptosis mechanism of recombinant ORM2 protein in distant organs, we added recombinant ORM2 protein to the culture medium of human embryonic kidney 293 T cells (which do not express ORM2). Immunofluorescence showed that recombinant ORM2 was taken up in 293 T cells, with or without RSL3 treatment (Fig. 7E). Western blot analysis also confirmed the uptake of rORM2 by 293 T cells (Fig. 7F). Moreover, the addition of rORM2 reduced ferrous ion levels, oxidative stress, and decreased the sensitivity of 293 T cells to ferroptosis (Fig. 7G–H and Fig. S7A–E). Importantly, endocytosis inhibitors effectively blocked the protective effects of ORM2 (Fig. 7H and Fig. S7A-S7E). To further investigate ORM2 internalization mechanisms, we employed pharmacological inhibitors targeting distinct endocytic pathways: Amiloride (macropinocytosis inhibitor), Filipin (caveolae-mediated endocytosis inhibitor), and Dynasore (clathrin-mediated endocytosis inhibitor). Our findings reveal that exogenous ORM2 uptake occurs primarily through macropinocytosis under basal conditions (Amiloride-sensitive but Dynasore-insensitive), while ferroptotic stress (FINO2 treatment) triggers engagement of both macropinocytosis and dynamin-dependent (likely clathrin-mediated) pathways (inhibited by both Amiloride and Dynasore) (Fig. S8A). These findings demonstrate a context-dependent shift in ORM2 internalization mechanisms: under basal conditions, ORM2 enters cells primarily through non-specific endocytosis (micropinocytosis), while ferroptotic stress induces a transition to receptor-mediated endocytosis as the dominant uptake pathway. This dynamic regulation suggests that stress conditions may activate specific receptor systems to facilitate more efficient ORM2 uptake, potentially enhancing its cytoprotective function during ferroptosis. Overall, these findings indicate that administered ORM2 protein can be absorbed by cells other than hepatocytes, allowing it to exert its anti-ferroptosis effect at the cellular level across multiple organs injuries (Fig. 7I).

## Disscussion

In this study, we identified that ORM2, a hepatokine secreted during acute injury, mitigates tissue damage by suppressing ferritinophagy, thereby reducing ferrous iron accumulation and ferroptosis. Treatment with recombinant ORM2 protein protects against APAP-induced liver injury and ischemia-reperfusion injuries in the liver and other tissues (Fig. 7I). These findings highlight ORM2’s function as a rapid response molecule that maintains cellular homeostasis and its potential as a therapeutic agent for acute tissue injury.

Acute phase response proteins, mainly synthesized by the liver during injury or inflammation, usually serve as blood markers. We found that ORM2 also plays critical role in reducing ferritinophagy and protecting against tissue damage. While previous studies have implicated ORM2 in inflammation [36, 52], the current study utilized a single-dose administration protocol with tissue collection within 24 h, during which no adverse effects were observed. However, we acknowledge that long-term safety assessments warrant future investigation. The comprehensive preclinical evaluation, including dose-response studies, chronic administration experiments, pharmacokinetic profiling (e.g., half-life of the recombinant protein), multi-organ toxicity assessments, and monitoring of iron metabolism parameters (e.g., serum iron, ferritin, total iron-binding capacity, and hemoglobin), will be essential for therapeutic development and mitigate potential iron deficiency risks.

The family member ORM1 has a similar molecular structure of ORM2 and is also an acute phase response protein under physiological conditions. However, it is unclear whether ORM1 has similar or complementary functions to ORM2. In our experiments, we found that overexpressing ORM1 does not protect against ferroptosis (Fig. S1F–S1G), indicating that ORM2 and ORM1 have different roles. However, the effects of ORM1 on other forms of cell death have not yet been studied. It is also necessary to explore its role in injury using ORM1 knockout mice or ORM1/ORM2 double knockout mice.

In this study, we show that ORM2 is dramatically upregulated and acts as a suppressor of ferritinophagy, implying that the endogenous ORM2 response may determine ferroptosis resistance in the liver under stress. However, under basal conditions, given that ORM2 is a liver secretory protein, we speculate that the mild baseline secretion of ORM2 may help maintain basal iron homeostasis in hepatocytes and prevent abnormal iron accumulation through some autocrine/paracrine mechanism. This “housekeeping” function may lay the foundation for its more robust protective role under stress conditions.

Multiple studies and reviews have confirmed that signs of ferritinophagy and ferroptosis activation can be observed in the liver tissues of patients with DILI, indicating its significant clinical relevance in the development and progression of human DILI [21, 22, 53]. Similar exploration in one study, the heparin derivative Sevuparin demonstrate that pharmacologically inhibiting the core iron-regulatory hormone hepcidin (via the BMP/SMAD pathway) can effectively improve iron mobilization and attenuate inflammation, with efficacy shown from murine models to healthy humans [54]. This example underscores the therapeutic potential of precisely modulating iron homeostasis. Additionally, we analyzed serum samples from patients with acute liver or kidney injury. Western blot results revealed elevated ORM2 expression and MDA levels, suggesting a potential link between ORM2 and ferroptosis under injury conditions (revised Fig. S8B–C). However, due to the limited sample availability, we could not establish definitive ORM2 levels or a cutoff threshold for disease prediction in this study. Future research should explore the specific manifestations and clinical significance of ORM2, particularly as an upstream regulator in human DILI, as this remains an unresolved gap in the field.

In our study, we demonstrate that ORM2 administration, both pre- and post-treatment, significantly reduces liver damage following APAP exposure. Notably, compared to the traditional antioxidant NAC, ORM2 exhibits much greater efficacy, suggesting its potential target for clinical translation as a therapeutic agent for chemically induced liver injury. However, our study exclusively utilized male mice, we have conducted additional experiments to investigate whether ORM2 infusion therapy also exerts protective effects in female mice. Surprisingly, in contrast to the strong protective effect observed in male mice, the administration of recombinant ORM2 protein failed to confer protection in females (Fig. S8D–F). This sexual dimorphism may be explained by the established intrinsic resistance of female hepatocytes to ferroptosis, which is associated with higher basal FTH1 expression [55].

In this study, we focused on apoptosis, necroptosis, and ferroptosis—three canonical cell death pathways in APAP‑induced liver injury. Pyroptosis (via NLRP3/caspase‑1/GSDMD) was not evaluated, as its kinetics and detection methods differ substantially. Whether ORM2 modulates pyroptosis remains to be explored in the future. In addition, while our mechanistic studies support the role of ORM2 in inhibiting ferroptosis, we acknowledge that the inhibitors DFO and Fer-1, though widely used, may have pleiotropic effects beyond ferroptosis suppression. Future studies could employ more specific pharmacological tools (e.g., Liproxstatin-1) or genetic models targeting core ferroptosis components, and such approaches would help to further solidify the specificity of this cell death pathway in our model and the precise mechanism of ORM2 [56]. Moreover, using biochemical method, we identified that ORM2 competitively binds to the N-terminal region (aa1-383) of NCOA4, displacing TAX1BP1—though the structural details of this interaction remain unresolved. Future studies employing crystallography, cryo-EM, or AI-assisted molecular docking could define the precise ORM2-NCOA4 binding interface, providing critical insights for ferroptosis intervention.

Although our study has not yet identified specific ORM2 receptors in hepatic or injured tissues, this remains a crucial research direction for understanding its ferroptosis-protective mechanisms. Notably, glypican family receptors (e.g., GYPC) have been identified as ORM2-binding partners in rheumatoid arthritis [57–59]. Therefore, validating the known or identify a novel receptor-ligand interaction and its functional consequence will be essential for elucidating ORM2’s cytoprotective actions and developing therapeutic targets for ferroptosis-associated diseases.

## Materials and Methods

### Cell lines

HEK293T cells (human embryonic kidney, male), HepG2 cells (human hepatocellular carcinoma, male), AML12 (mouse liver) were cultured in Dulbecco’s Modified Eagle Medium (DMEM, L110KJ), with supplemented with 100 U/mL penicillin and 100 μg/mL streptomycin (S110JV) and 10% (v/v) fetal bovine serum (FBS, S760JY). The cultures were maintained in a humidified atmosphere containing 5% CO₂ at 37°C.

### Human Samples

In Fig. S8B–C, the serum samples were collected from five patients with acute liver or kidney injury, along with samples from healthy volunteers. Prior informed consent was obtained from each patient, and the research was approved by the ethics committee of Shanghai Jiao Tong University School of Medicine. The Ethics Committee of First Affiliated Hospital, Shanghai Jiao Tong University School of Medicine approved the collection and use of human samples (2020KY051).

### Plasmid construction

*Flag-ORM2, HA-ORM2, Flag-TAX1BP1, Myc-NCOA4* were cloned into pLVX lentiviral vector. sh*ORM2* were cloned into pGIPZ lentiviral vector. *EGFP* was cloned into PT3 transposon vector.

### Reagents

The reagents used: 1S,3R-RSL3 (Selleck, S8155), FINO2 (Selleck, E1244), Amiloride hydrochloride (MedChem Express, HY-B0285A), Ferrostatin-1 (Fer-1) (Selleck Chemicals, S7243), Deferoxamine mesylate (DFO) (MedChem Express, HY-B0988). Cell Ferrous Iron (Fe^2+^) Fluorometric Assay Kit (Meilunbio, MA0647), 4-Hydroxynonenal Antibody (R&D Systems, MAB3249), NAC (Beyotime, S0077). Human ORM2 protein (MedChem Express, HY-P7490). Mouse ORM2-Fc protein (Fc region from mouse IgG) was generated at Changzhou Smart-Lifesciences Biotechnology using a mammalian expression system (Expi293 cells) and purified by Ni-NTA purification system.

### Primary hepatocyte isolation and culture

Primary hepatocytes were isolated by two-step collagenase perfusion as previously reported [60]. Liver perfusion medium (Thermo Fisher Scientific, 17701038), liver digest medium (Thermo Fisher, 17703034), Hepatocyte wash medium (Thermo Fisher, 17704024) and rat tail collagen (Corning, 354236) were used.

### Generation of *ORM2* overexpression stable cell line

The pLVML-*Flag-ORM2* lentiviral plasmids with the packaging plasmids pMD2.G and psPAX2 were co-transfected into HEK293T cells. Lenti-viral particles were collected and filtered through a 0.45-μm filter (BIOFIL, FPE404030). The filtrate was used to infect HepG2 and AML12 cells in the presence of 10 μg/mL polybrene (Yeasen, 40804ES76). Stable cell lines were selected using 1 μg/mL puromycin (Beyotime, ST551).

### Generation of *ORM2* knockdown stable cell line

Two sequences targeting human *ORM2* were cloned into the pGIPZ lentiviral vector: sh1-CAGGTCAGATGTCATGTAC and sh2-CCAGAACCAGTGCTTCTAT. Additionally, two sequences targeting TAX1BP1 were used: sh1-AGATCAATCAGCTAATAAT and sh2-AGAAGATACTTGTTTTTTA. Stable cell lines were established using the aforementioned lentiviral packaging and transduction methods.

### Cell viability and cell death detection

Cell viability was assessed using the CCK-8 kit (MCE, HY-K0301). 8 × 10^3^ primary hepatocytes or HepG2 cells were seeded per well in a 96-well plate. For AML12 cells, 2 × 10^4^ cells were seeded per well. Cell death was assessed using propidium iodide (PI) staining (LianKe Bio, AP107-30).

### Superoxide anions (ROS) detection by DCFH-DA and DHE probes

Cells were seeded in 6-well plates at a density of 1.2 × 10^6^ cells per well. Following treatment with RSL3 or FINO2 for 4 h, cells were incubated with 0.5 µM DHE or 5 µM DCFH-DA dye for 30 min. Fluorescence microscopy was then performed, using excitation wavelengths of 535 nm for DHE and 488 nm for DCFH-DA.

### Mice

Wild-type C57BL/6 mice (8 to 10-week-old) were purchased from Shanghai SLAC Co. Ltd (Shanghai, China). C57BL/6 strain background *Orm2* knockout mice were generated in BIOCYTOGEN using CRISPR-Cas9 by targeted deletion of exon 1-exon 6 to produce whole-body knockout animals, following the same methodology described in our previous report (Fig. 1) [37]. Genotyping was performed using previously reported PCR primers. All experiments were done in an age-controlled fashion. For studies involving comparison between experimental groups, animals were randomly allocated to each group. Unless otherwise stated, all experiments utilized male mice. The sample size for each experimental group was chosen based on common practice in the field and our preliminary data to ensure reproducible observation of the phenotype. All animal experiments were approved by the Institutional Animal Care and Use Committee of Shanghai Jiao Tong University School of Medicine (Permit number: A-2021-012).

### Chemical liver injury experiments

According to the body surface area-based dose conversion principle (FDA guidance), a dose of 300 mg/kg APAP in mice is approximately equivalent to ~24 mg/kg (or about 1500 mg for a 60 kg adult) in humans. This dose significantly exceeds the conventional maximum daily therapeutic dose for adults (4 g), as it is intended to model acute and severe liver injury, corresponding to the pathological stage of clinical acetaminophen overdose [61]. After overnight fasting, mice were intraperitoneally injected with acetaminophen (APAP, APExBIO, B3532) at a dose of 300 mg/kg for liver injury or 750 mg/kg for liver failure according to literatures [62–65]. The mice were sacrificed after 6, 12, or 24 h. For Carbon Tetrachloride (CCl_4_) experiment, 10% CCl_4_ in corn oil were intraperitoneally injected at a dose of 0.5 ml/kg of mouse [65]. In the therapeutic model involving recombinant ORM2 protein infusion, mice were administered 7 mg/kg or 20 mg/kg of rORM dissolved in PBS prior to the induction of acute liver injury or ischemia-reperfusion injury. In the therapeutic model using DFO, mice received 200 mg/kg of DFO dissolved in PBS before acute liver injury was induced.

### Ischemia-Reperfusion (I/R) injury mouse models

Mice were anesthetized with isoflurane using anesthesia vaporizer (RWD) and placed on a heating pad to maintain a body temperature of 37 °C. For the 70% hepatic ischemia-reperfusion, we followed previously reported methods [66]. Briefly, atraumatic vascular clamps were applied to the portal vein, hepatic artery, and bile duct supplying the median and left lobes of the liver, resulting in 70% ischemia and was maintained for 1 h. Then the clamps were removed to initiate reperfusion for 12 h. For the renal ischemia-reperfusion model, the surgery was established as previously reported [67]. An incision was made 1 cm from the midline of the back to expose the unilateral renal artery, which was clamped for 45 min. Mice in the sham group underwent the same procedure without renal artery clamping. Mice were euthanized 16 h post-I/R, and kidney samples were collected for mRNA analysis, western blot analysis or paraffin embedding. To model acute lung injury induced by intestinal ischemia-reperfusion, we followed the established methods [68]. A midline laparotomy was performed to expose the intestines. The superior mesenteric artery (SMA) was identified and temporarily occluded using a microvascular clamp to induce ischemia for 45 min. Following the ischemic period, the clamp was removed to allow for reperfusion, which lasted for 3 h.

### Detection of indicators of tissue damage

Mouse blood was collected, and serum was used for alanine aminotransferase (ALT), aspartate aminotransferase (AST) (Nanjing Jiancheng, C009-2-1 and C010-2-1), creatinine (Cr) and blood urea nitrogen (BUN) (Nanjing Jiancheng, C011-2-1 and C013-2-1) analysis.

### Biochemical measurement

The levels of total glutathione (T-GSH) and oxidized glutathione (GSSG) in liver tissue homogenates were measured using the T-GSH/GSSG assay kit (Vazyme, A061-1-1). NAPQI levels in liver cells were assessed using an ELISA kit (Camilo Biological, 2M-KMLJM221060m). The activities of glutathione S-transferase (GST) and glutathione reductase (GR) in mouse liver samples were determined using kits (Soleilbio, BC0355 for GST and BC1165 for GR) according to the manufacturer’s instructions.

### FerroOrange staining of mouse tissue frozen sections

Freshly collected mouse tissues were embedded in OCT compound and rapidly frozen in liquid nitrogen for 2 min. Samples were then equilibrated in a cryostat at −20 °C for 2 h before sectioning. Tissue sections (5 μm thick) were mounted onto glass slides and air-dried for 15 min. After rinsing with PBS, sections were incubated with FerroOrange working solution (Meilunbio, MA0647) at room temperature for 30 mins. Following staining, slides were coverslipped and imaged using a Leica SP8X confocal microscope.

### Iron content, MDA and LPO measurement

Iron content in mouse tissues and cell lines were determined using tissue iron assay kit (Nanjing Jiancheng, A003-1-2) according to the instructions. Malondialdehyde (MDA) using the MDA assay kit (APExBIO, K2167), or the Lipid peroxidation assay kit (Nanjing Jiancheng, A106-1-3).

### Detection and imaging of labile iron

Intracellular ferrous iron (Fe^2+^) was detected using the FerroOrange probe (Dojindo, F374). After 4-h treatment with RSL3 or FINO2, cells were washed with and resuspended in HBSS containing FerroOrange for 15 min, samples were analyzed by flow cytometry (CytoFLEX LX, USA) or microscope.

### Histology, immunohistochemistry (IHC), and TUNEL Assay

Mouse liver, kidney and lung tissues were fixed in 4% paraformaldehyde (PFA) overnight and then embedded in paraffin. Sections were stained with hematoxylin and eosin according to H&E staining protocol. For IHC, primary antibodies used were Cytochrome P450 2E1 antibody (Abcam, ab28146), Cleaved caspase-3 (Abcam, 9661S), ORM2 (Abcam, ab231906), GFP (Abmart, M20004), and Ferritin Heavy Chain (Abclone, A25458). Detection was performed with Elite ABC Kit and DAB Substrate (Vector Laboratories, ZF1120 and SK4105). TUNEL staining was performed using the Vazyme kit (Vazyme, A112-02). Five random images were captured for each section, with a minimum of three mice per group. Images were analyzed using ImageJ.

### Immunoblotting (IB) and Co-immunoprecipitation (co-IP)

Tissue samples were lysed using Triton X-100 lysis buffer (50 mM Tris, 150 mM NaCl, 1% Triton X-100, pH 7.5) containing protease inhibitors (Roche, 4693132001) and phosphatase inhibitors (Bimake, B15002). Antibodies used for IB: Vinculin (Cell Signaling Technology, 13901S), ORM2 (Abcam, ab231906), Cleaved Caspase-3 (Abclone, 9661), Transferrin Receptor (ABclonal, A5865), GPX4 (Abclone, A11243), FSP1 (ProteinTech, 20886-1-A), DHODH (ProteinTech, 14877-1-AP), SLC7A11 (Thermo Fisher Scientific, PA1-16893), GAPDH (Abmart, M20006S), FTL (ProteinTech, 10727-1-AP), FTH (Abclone, A25458), Flag (Sigma-Aldrich, F1804), Myc (Abclonal, AE070), HA (Cell Signaling Technology, 3724), NCOA4 (HUABIO, HA722282) and TAX1BP1 (HUABIO, HA721648). Antibodies or beads used for co-IP: FTL (ProteinTech, 10727-1-AP), FLAG beads (GenScript, L00432), Myc beads (Smart-lifesciences, SA065005), and protein A/G magnetic beads (CYTOCH, PM0004).

### Immunofluorescence (IF)

IF were performed according to the previously reported method [65]. Antibodies used for IF: FTL (ProteinTech, 10727-1-AP), FTH (Abclone, A25458), TAX1BP1 (Proteintech, 14424-1-AP), LAMP1 (Cell Signaling Technology, 9091), LAMP2 (ProteinTech, 66301-1-Ig), and ORM2 (R&D Systems, 959445). After washing with PBS, the cells were incubated with fluorophore-conjugated secondary antibodies (Invitrogen, A11008 and A11006). Following additional washes, the samples were mounted using an anti-fade mounting medium (Absin, abs9235). Imaging was performed using a Zeiss LSM 980 confocal microscope.

### RNA extraction and RT-qPCR

Total RNA was extracted from cells using the TIANGEN kit (DP419) following the manufacturer’s instructions. Equal amounts of RNA (1 μg) were reverse transcribed into cDNA using the Vazyme kit (R323-01). cDNA was diluted and subjected to qPCR using the Vazyme SYBR Green Premix Pro Taq HS qPCR Kit (Q771-02). The primer sequences are in the supplemental Table S1.

### RNA sequencing

Total RNA was extracted from WT and *Orm2*^*-/-*^ mouse tissues 24 h post-APAP treatment using the miRNeasy Mini Kit (Qiagen, 74104). mRNA libraries were constructed with Illumina kit (NEB, E7775S). All RNA-seq libraries were sequenced on the NovaSeq 6000 platform (Illumina) for 150-bp paired-end sequencing.

### RNA-Seq analysis

Raw FASTQ files underwent quality control and adapter trimming with fastp. Processed reads were aligned to the mouse reference genome (GRCm38.p6) using HISAT2 [69], and raw counts were generated with HTSeq-count [70]. FPKM values for each sample were calculated using StringTie. Differential gene expression analysis and heatmap generation were performed using edgeR in R Studio [71]. Genes exhibiting a fold change (|FC | ) > 1.5 and *P* < 0.05 were classified as differentially expressed.

### Statistical analysis

The experiments and analysis were conducted blindly and replicated at least three times independently. Statistical analyses were performed using GraphPad Prism 9.0 software. Experimental data are presented as mean ± standard error of the mean (SEM) for each independent experiment. For comparisons between two groups, an unpaired, two-tailed Student’s t-test was used. Comparisons among multiple groups were analyzed by one-way ANOVA followed by Dunnett’s multiple comparisons test. For data involving two independent variables, two-way ANOVA was applied, followed by Bonferroni’s post-hoc test. The significance of survival differences was determined by the log-rank (Mantel–Cox) test. The significance levels were set at *P < 0.05, **P < 0.01, ***P < 0.001 and ****P < 0.0001.

## Supplementary information

Supplemental Figures and Tables

original western blots

## Data Availability

All data are available upon request from the corresponding author.
