# Supplementary material for: Hepatokine ORM2 suppresses pathological ferritinophagy to prevent acute tissue injury
Source: Cell Death Dis. 2026 Apr 30;17(1):578. doi: 10.1038/s41419-026-08803-0 (PMC13275894; doi:10.1038/s41419-026-08803-0)
Supplement: Supplementary file 1 — Supplemental Figures and Tables [file 41419_2026_8803_MOESM1_ESM.pdf]

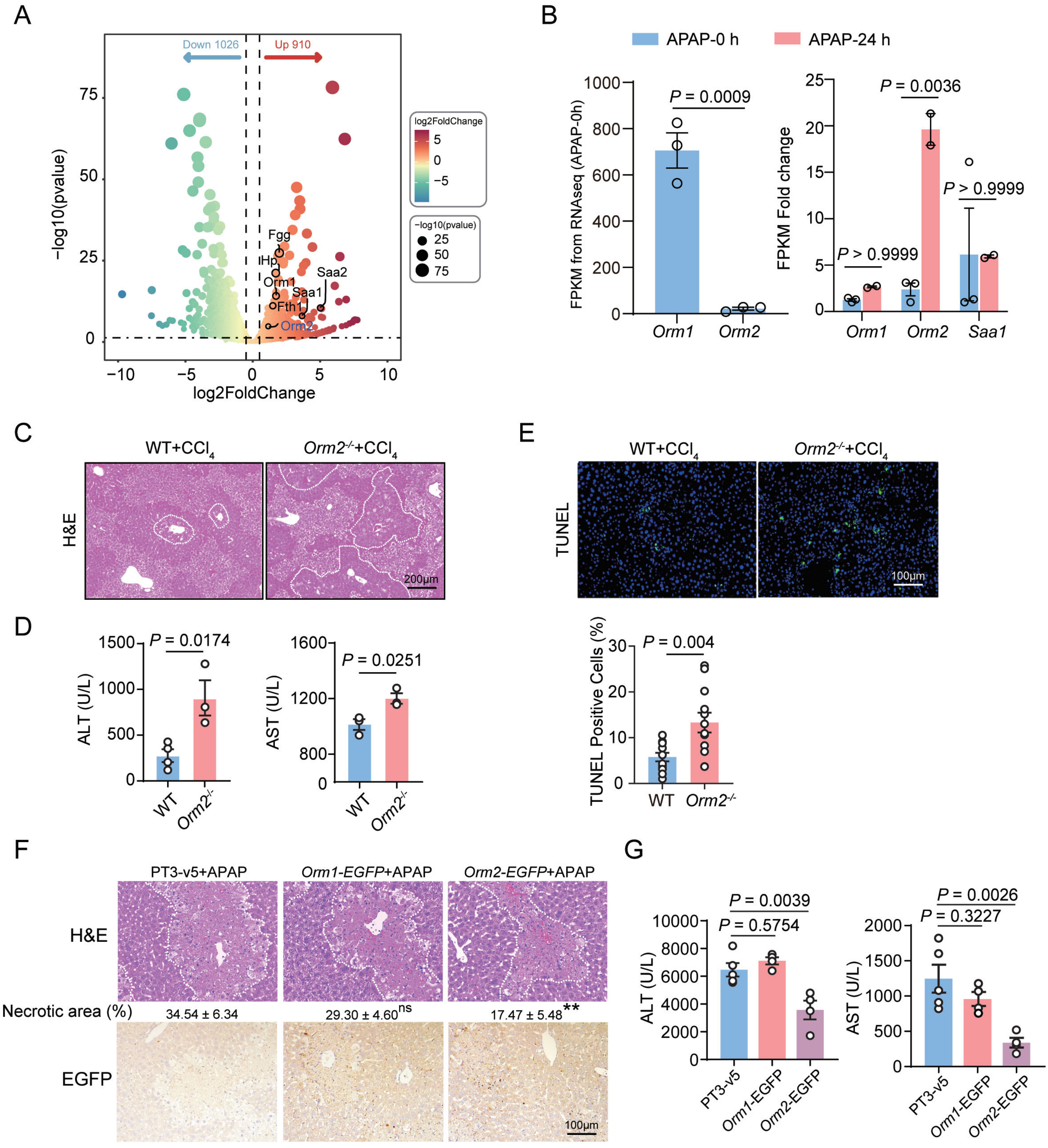

A
Down 1026
Up 910
-log10(pvalue)
log2FoldChange
Fgg
Hp
Orm1
Saa2
Saa1
Fth1
Orm2
B
APAP-0 h
APAP-24 h
FPKM from RNAseq (APAP-0h)
P = 0.0009
Orm1
Orm2
FPKM Fold change
P = 0.0036
P > 0.9999
P > 0.9999
Saa1
C
WT+CCl4
Orm2-/-+CCl4
H&E
200μm
D
ALT (U/L)
P = 0.0174
AST (U/L)
P = 0.0251
WT
Orm2-/-
E
TUNEL
100μm
TUNEL Positive Cells (%)
P = 0.004
F
PT3-v5+APAP
Orm1-EGFP+APAP
Orm2-EGFP+APAP
H&E
Necrotic area (%)
34.54 ± 6.34
29.30 ± 4.60ns
17.47 ± 5.48**
EGFP
100μm
G
P = 0.0039
P = 0.5754
P = 0.0026
P = 0.3227
PT3-v5
Orm1-EGFP
Orm2-EGFP

Figure S1

Figure S1. Loss of *Orm2* in hepatocytes exacerbates acute liver injury in mice

(A) Differential gene expression analysis of RNA-seq from livers of 24 hours post-APAP injection, comparing APAP-treated mouse liver to solvent-injected controls, based on sequencing data from Shani Ben-Moshe et al. (2022). The volcano plot displays log2 fold change versus -log10 p-value for each gene, highlighting significant gene upregulation and downregulation in response to APAP treatment. Genes with $p < 0.05$ are marked, indicating statistically significant expression changes.

(B) Gene expression using RNA-seq data from livers collected 24 hours post-APAP injection, comparing APAP-treated mouse liver (n=2) to solvent-injected controls (n=3).

(C) WT and *Orm2*$^{-/-}$ mice were injected with 10% CCl_4 at a dose of 5 μL/g body weight and examined 24 hours later. Representative H&E images of liver tissues from WT and *Orm2*$^{-/-}$ mice. Necrotic areas circled with white lines (n = 4 per group). Scale bar, 200 μm.

(D) Serum ALT and AST in WT and *Orm2*$^{-/-}$ mice from **(C)**.

(E) TUNEL staining of livers from WT and *Orm2*$^{-/-}$ mice in **(C)**. Quantification on the right. Scale bar, 100 μm.

(F) Sleeping beauty transposon constructs (PT3-*Vector*, PT3-*Orm1-EGFP*, or PT3-*Orm2-EGFP*) together with SB100 transposase were delivered into mice via hydrodynamic tail vein injection (HDT), followed one week later by APAP administration; EGFP-ORM1/ORM2 expression in liver sections was assessed by immunohistochemistry and intrahepatic staining with quantification (below) of H&E was performed. Necrotic areas circled with white lines (n = 4-5 per group). Scale bar, 100

μm.

(G) Serum ALT and AST in mice with overexpression of PT3-*Vector*, PT3-*Orm1-EGFP*, or PT3-*Orm2-EGFP* from **(F)**.

All data in this figure are represented as mean ± SEM. In (B), data were analyzed by two-way ANOVA followed by Bonferroni's multiple comparisons test; in (F) and (G), by one-way ANOVA with Dunnett's test; the remaining data were analyzed by unpaired two-tailed Student's *t* test. Exact *p* values are provided in the figure. "n" refers to biological replicates. All experiments were performed in triplicates.

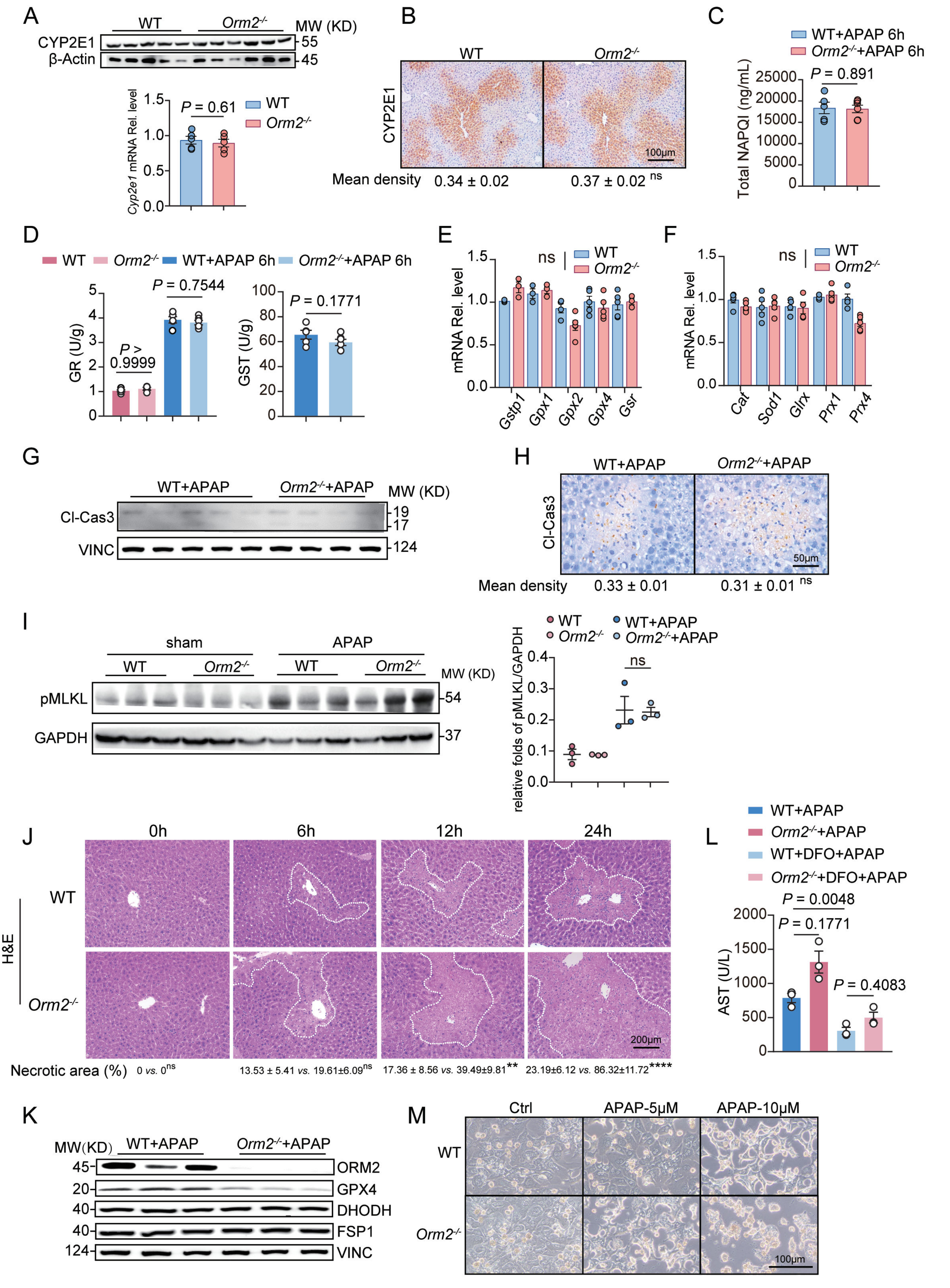
A
WT
Orm2-/-
MW (KD)
CYP2E1
β-Actin
55
45
Cyp2e1 mRNA Rel. level
P = 0.61
WT
Orm2-/-
B
WT
Orm2-/-
CYP2E1
100μm
Mean density 0.34 ± 0.02 0.37 ± 0.02 ns
C
WT+APAP 6h
Orm2-/-+APAP 6h
Total NAPQI (ng/mL)
P = 0.891
D
WT Orm2-/- WT+APAP 6h Orm2-/-+APAP 6h
GR (U/g)
P > 0.9999
P = 0.7544
GST (U/g)
P = 0.1771
E
mRNA Rel. level
ns
WT
Orm2-/-
Gstp1 Gpx1 Gpx2 Gpx4 Gsr
F
mRNA Rel. level
ns
WT
Orm2-/-
Cat Sod1 Glrx Prx1 Prx4
G
WT+APAP
Orm2-/-+APAP
MW (KD)
Cl-Cas3
VINC
19
17
124
H
WT+APAP
Orm2-/-+APAP
Cl-Cas3
50μm
Mean density 0.33 ± 0.01 0.31 ± 0.01 ns
I
sham
APAP
WT
Orm2-/-
WT
Orm2-/-
MW (KD)
pMLKL
GAPDH
54
37
WT
Orm2-/-
WT+APAP
Orm2-/-+APAP
relative folds of pMLKL/GAPDH
ns
J
0h 6h 12h 24h
WT
Orm2-/-
H&E
200μm
Necrotic area (%) 0 vs. 0ns 13.53 ± 5.41 vs. 19.61±6.09ns 17.36 ± 8.56 vs. 39.49±9.81** 23.19±6.12 vs. 86.32±11.72****
L
WT+APAP
Orm2-/-+APAP
WT+DFO+APAP
Orm2-/-+DFO+APAP
AST (U/L)
P = 0.0048
P = 0.1771
P = 0.4083
K
MW(KD)
WT+APAP
Orm2-/-+APAP
45
20
40
40
124
ORM2
GPX4
DHODH
FSP1
VINC
M
Ctrl
APAP-5μM
APAP-10μM
WT
Orm2-/-
100μm

Figure S2

Figure S2. *Orm2* deficiency does not affect the intracellular metabolism of APAP

(A) The protein and mRNA levels of *Cyp2e1* in liver tissues from WT and *Orm2*$^{-/-}$ mice at baseline (n = 6 per group).

(B) Representative CYP2E1 immunohistochemistry (IHC) staining of liver sections from WT and *Orm2*$^{-/-}$ mice at baseline. Scale bar, 100 μm.

(C) Quantification of NAPQI in liver tissues from WT and *Orm2*$^{-/-}$ mice 6 hours after injection with 300 mg/kg APAP (n = 6 per group).

(D) Enzyme activity of GR and GST in liver tissues from WT and *Orm2*$^{-/-}$ mice before and after injection with 300 mg/kg APAP (n = 6 per group).

(E-F) mRNA levels of major phase II metabolic enzymes and antioxidant enzymes in liver tissues from WT and *Orm2*$^{-/-}$ mice at baseline, measured by qPCR (n = 6 per group).

(G) Cleaved caspase-3 protein levels of liver tissues from WT and *Orm2*$^{-/-}$ mice 24 hours post-APAP, measured by western blot.

(H) Representative cleaved caspase-3 IHC staining of liver tissues from WT and *Orm2*$^{-/-}$ mice 24 hours post-APAP. Scale bar, 50 μm. The average density of positive staining areas was quantified.

(I) Phospho-MLKL protein levels of liver tissues from WT and *Orm2*$^{-/-}$ mice 24 hours post-APAP, measured by western blot. Quantification is on the right.

(J) WT and *Orm2*$^{-/-}$ mice received APAP (300 mg/kg), liver samples were collected at the indicated time points. Representative H&E staining showing liver damage; damaged areas are outlined by white dashed lines. Quantification of the percentage of

damaged area is shown below. Scale bars, 200 μm.

(K) Liver tissue samples from WT and *Orm2*$^{-/-}$ mice, 24 hours post-APAP treatment, western blot analysis of ORM2, GPX4, DHODH, and FSP1 protein levels.

(L) WT and *Orm2*$^{-/-}$ mice were pre-treated with 200 mg/kg DFO or vehicle, then injected with APAP for 24 hours. Serum AST levels from mice in were measured.

(M) Primary hepatocytes isolated from WT and *Orm2*$^{-/-}$ mice were treated with APAP. Representative bright-field images revealed dose-dependent cell death phenotypes including cell rounding and detachment during APAP-treatment, with more pronounced effects in ORM2-deficient cells. Scale bar, 100 μm.

To assess early metabolic response and late cell death activation respectively, GST/GR activities and NAPQI adducts were measured at 6 h post-APAP (C, D); cleaved caspase-3 and pMLKL levels were examined at 24 h post-APAP (G, I).

All data in this figure are represented as mean ± SEM. Data were analyzed by two-way ANOVA followed with Bonferroni's post hoc test for comparisons between genotypes under different conditions; the remaining data were analyzed by unpaired two-tailed Student's *t* test. Exact *p* values are provided in the figure. $^{**}p < 0.01$, $^{****}p < 0.0001$, and ns (no significance). "n" refers to biological replicates. All experiments were performed in triplicates.

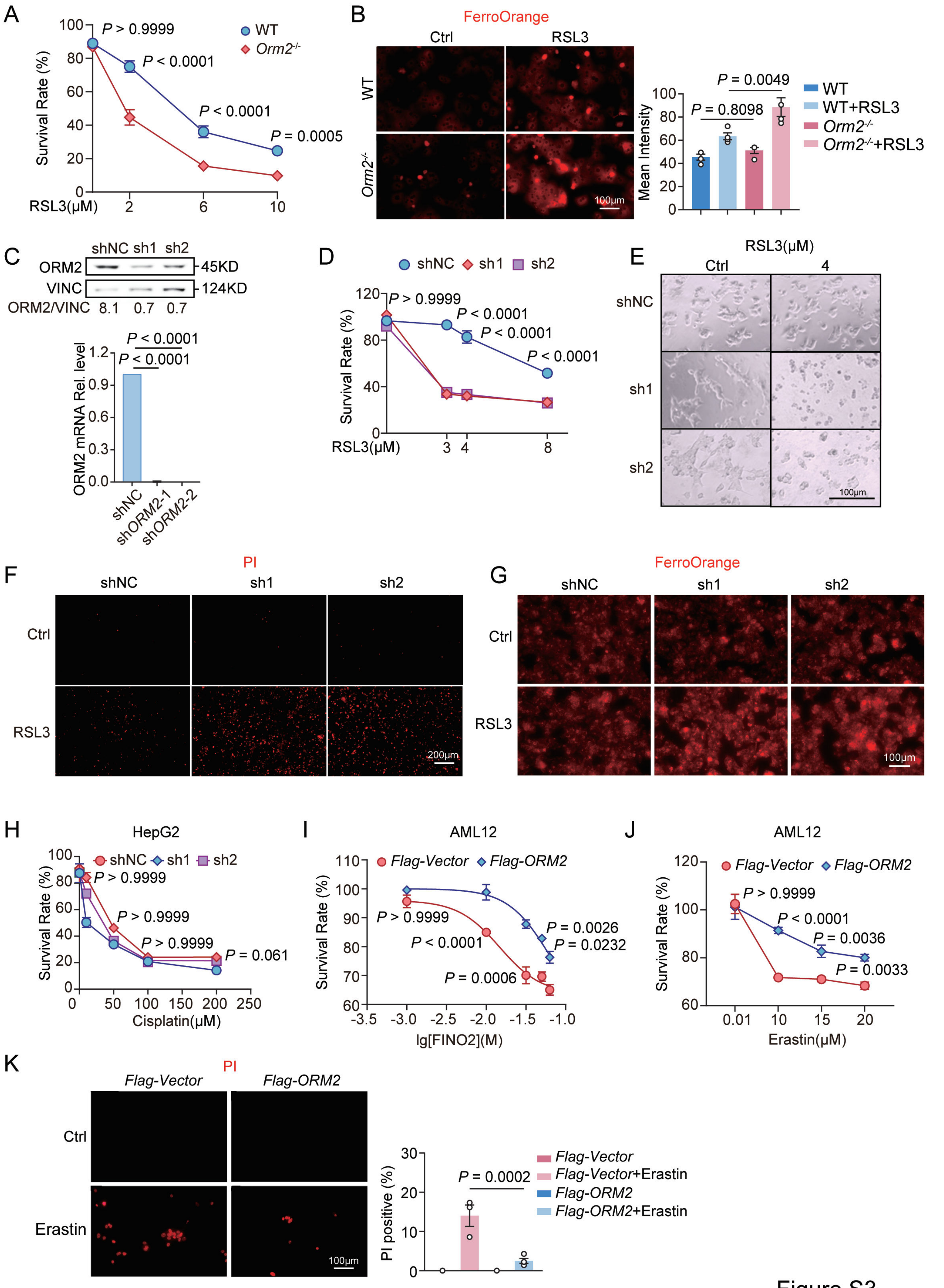
A
P > 0.9999
P < 0.0001
P < 0.0001
P = 0.0005
WT
Orm2-/-
Survival Rate (%)
RSL3(μM)
B
FerroOrange
Ctrl
RSL3
WT
Orm2-/-
100μm
Mean Intensity
P = 0.0049
P = 0.8098
WT
WT+RSL3
Orm2-/-
Orm2-/-+RSL3
C
shNC sh1 sh2
ORM2
VINC
45KD
124KD
ORM2/VINC 8.1 0.7 0.7
P < 0.0001
P < 0.0001
ORM2 mRNA Rel. level
shNC
shORM2-1
shORM2-2
D
shNC sh1 sh2
P > 0.9999
P < 0.0001
P < 0.0001
P < 0.0001
Survival Rate (%)
RSL3(μM)
E
RSL3(μM)
Ctrl
4
shNC
sh1
sh2
100μm
F
PI
shNC
sh1
sh2
Ctrl
RSL3
200μm
G
FerroOrange
shNC
sh1
sh2
Ctrl
RSL3
100μm
H
HepG2
shNC sh1 sh2
P > 0.9999
P > 0.9999
P > 0.9999
P = 0.061
Survival Rate (%)
Cisplatin(μM)
I
AML12
Flag-Vector
Flag-ORM2
P > 0.9999
P < 0.0001
P = 0.0006
P = 0.0026
P = 0.0232
Survival Rate (%)
lg[FINO2](M)
J
AML12
Flag-Vector
Flag-ORM2
P > 0.9999
P < 0.0001
P = 0.0036
P = 0.0033
Survival Rate (%)
Erastin(μM)
K
PI
Flag-Vector
Flag-ORM2
Ctrl
Erastin
100μm
PI positive (%)
P = 0.0002
Flag-Vector
Flag-Vector+Erastin
Flag-ORM2
Flag-ORM2+Erastin

Figure S3

Figure S3. ORM2 deficiency increases cellular sensitivity to ferroptosis inducers

(A) Primary hepatocytes from WT and *Orm2*$^{-/-}$ mice were treated with RSL3 at the indicated concentrations for 12 hours, and cell viability was assessed using CCK8.

(B) Primary hepatocytes from WT and *Orm2*$^{-/-}$ mice were treated with 6 μM RSL3 for 4 hrs. Fe^{2+} levels were visualized using FerroOrange staining. Scale bar, 100 μm. Quantification on the right.

(C) Knockdown efficiency of sh*ORM2* in HepG2 cells confirmed by immunoblotting and quantitative real-time PCR.

(D) *ORM2* stable knockdown and control HepG2 cells were treated with RSL3 at the indicated concentrations for 12 hours, and cell viability was assessed using the CCK8 assay.

(E) Representative bright-field images of *ORM2* stable knockdown and control HepG2 cells treated with 4 μM RSL3 for 12 hours. Scale bar, 100 μm.

(F) Representative PI staining images of *ORM2* stable knockdown and control HepG2 cells treated with 4 μM RSL3 for 12 hours. Scale bar, 200 μm.

(G) *ORM2* stable knockdown and control HepG2 cells were treated with 4 μM RSL3 for 12 hours. Fe^{2+} levels were visualized using FerroOrange staining. Scale bar, 100 μm.

(H) *ORM2* stable knockdown and control HepG2 cells treated overnight with cisplatin at the indicated concentrations, and cell viability were measured using the CCK8 assays.

(I) AML12 cells with stable *Flag-ORM2* overexpression or empty vector control, were

treated with the indicated concentrations of FINO2 for 12 hours, and cell viability was assessed using CCK8.

(J) AML12 cells with stable *Flag-ORM2* overexpression or empty vector control, were treated with the indicated concentrations of erastin for 12 hours, and cell viability was assessed using CCK8.

(K) Representative PI staining images of AML12 cells from **(J)** treated with 10 μM erastin for 12 hours; quantification of fluorescence intensity is shown on the right. Scale bar, 100 μm.

All data in this figure are represented as mean ± SEM. In (C), data were analyzed by unpaired two-tailed Student's *t* test; the remaining data were analyzed by two-way ANOVA followed with Bonferroni's post hoc test. Exact *p* values are provided in the figure. All experiments were performed in triplicates.

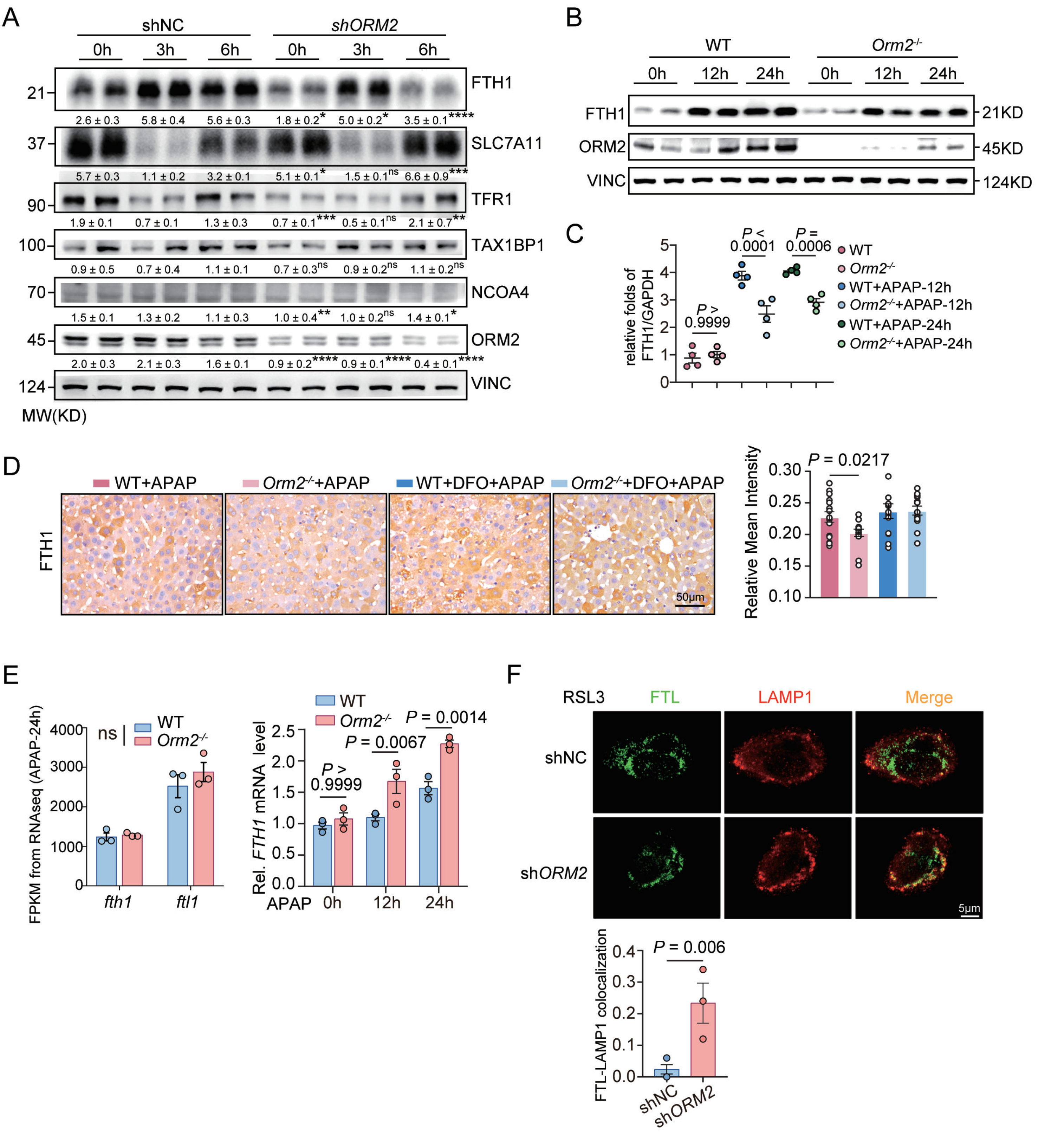
A
shNC
shORM2
0h
3h
6h
21
FTH1
2.6 ± 0.3
5.8 ± 0.4
5.6 ± 0.3
1.8 ± 0.2*
5.0 ± 0.2*
3.5 ± 0.1****
37
SLC7A11
5.7 ± 0.3
1.1 ± 0.2
3.2 ± 0.1
5.1 ± 0.1*
1.5 ± 0.1ns
6.6 ± 0.9***
90
TFR1
1.9 ± 0.1
0.7 ± 0.1
1.3 ± 0.3
0.7 ± 0.1***
0.5 ± 0.1ns
2.1 ± 0.7**
100
TAX1BP1
0.9 ± 0.5
0.7 ± 0.4
1.1 ± 0.1
0.7 ± 0.3ns
0.9 ± 0.2ns
1.1 ± 0.2ns
70
NCOA4
1.5 ± 0.1
1.3 ± 0.2
1.1 ± 0.3
1.0 ± 0.4**
1.0 ± 0.2ns
1.4 ± 0.1*
45
ORM2
2.0 ± 0.3
2.1 ± 0.3
1.6 ± 0.1
0.9 ± 0.2****
0.9 ± 0.1****
0.4 ± 0.1****
124
VINC
MW(KD)
B
WT
Orm2-/-
0h
12h
24h
FTH1
21KD
ORM2
45KD
VINC
124KD
C
relative folds of FTH1/GAPDH
P > 0.9999
P < 0.0001
P = 0.0006
WT
Orm2-/-
WT+APAP-12h
Orm2-/-+APAP-12h
WT+APAP-24h
Orm2-/-+APAP-24h
D
WT+APAP
Orm2-/-+APAP
WT+DFO+APAP
Orm2-/-+DFO+APAP
FTH1
50μm
Relative Mean Intensity
P = 0.0217
E
FPKM from RNAseq (APAP-24h)
ns
WT
Orm2-/-
fth1
ftl1
Rel. FTH1 mRNA level
P > 0.9999
P = 0.0067
P = 0.0014
APAP
0h
12h
24h
F
RSL3
FTL
LAMP1
Merge
shNC
shORM2
5μm
FTL-LAMP1 colocalization
P = 0.006
shNC
shORM2

Figure S4

Figure S4. ORM2 deficiency enhances ferritinophagy

(A) *ORM2* stable knockdown and control HepG2 cells were treated with 8 μM FINO2 for the indicated time periods. Representative immunoblots and quantitative analyses of FTH1, SLC7A11, TFR1, TAX1BP1, NCOA4, and ORM2 are shown.

(B) Western blot analysis of FTH1 and ORM2 in liver tissues. WT and *Orm2*$^{-/-}$ mice were injected with APAP (300 mg/kg), and liver samples were collected at the indicated time points.

(C) Representative quantification of FTH1 and ORM2 protein levels from **(B)** (n = 4 per group).

(D) WT and *Orm2*$^{-/-}$ mice were pre-treatment with DFO or left untreated for 2 hours before APAP injection. After APAP 24 hours, liver tissue samples were collected for IHC staining of FTH1. The right panel showing the statistical analysis of average optical density using Image J. Scale bar, 50 μm.

(E) WT and *Orm2*$^{-/-}$ mice were injected with APAP (300mg/kg), liver samples were collected at 24 hours for RNA-seq and qPCR. *fth1 and ftl* from liver tissues were analyzed (n = 3 per group).

(F) HepG2 cells with *ORM2* knockdown or control were treated with 4 μM RSL3. Representative immunofluorescence images display ferritin (FTL) and lysosomes (LAMP1 positive foci) localization. Quantification of co-localization was analyzed using Image J (below panel). Scale bars, 5 μm.

All data in this figure are represented as mean ± SEM. In (D) and (F), data were analyzed by unpaired two-tailed Student's *t* test; the remaining data were analyzed by

two-way ANOVA followed with Bonferroni's post hoc test. Exact p values are provided in the figure. *$p < 0.05$, **$p < 0.01$ and ***$p < 0.001$, ****$p < 0.0001$ and ns (no significance). All experiments were performed in triplicates.

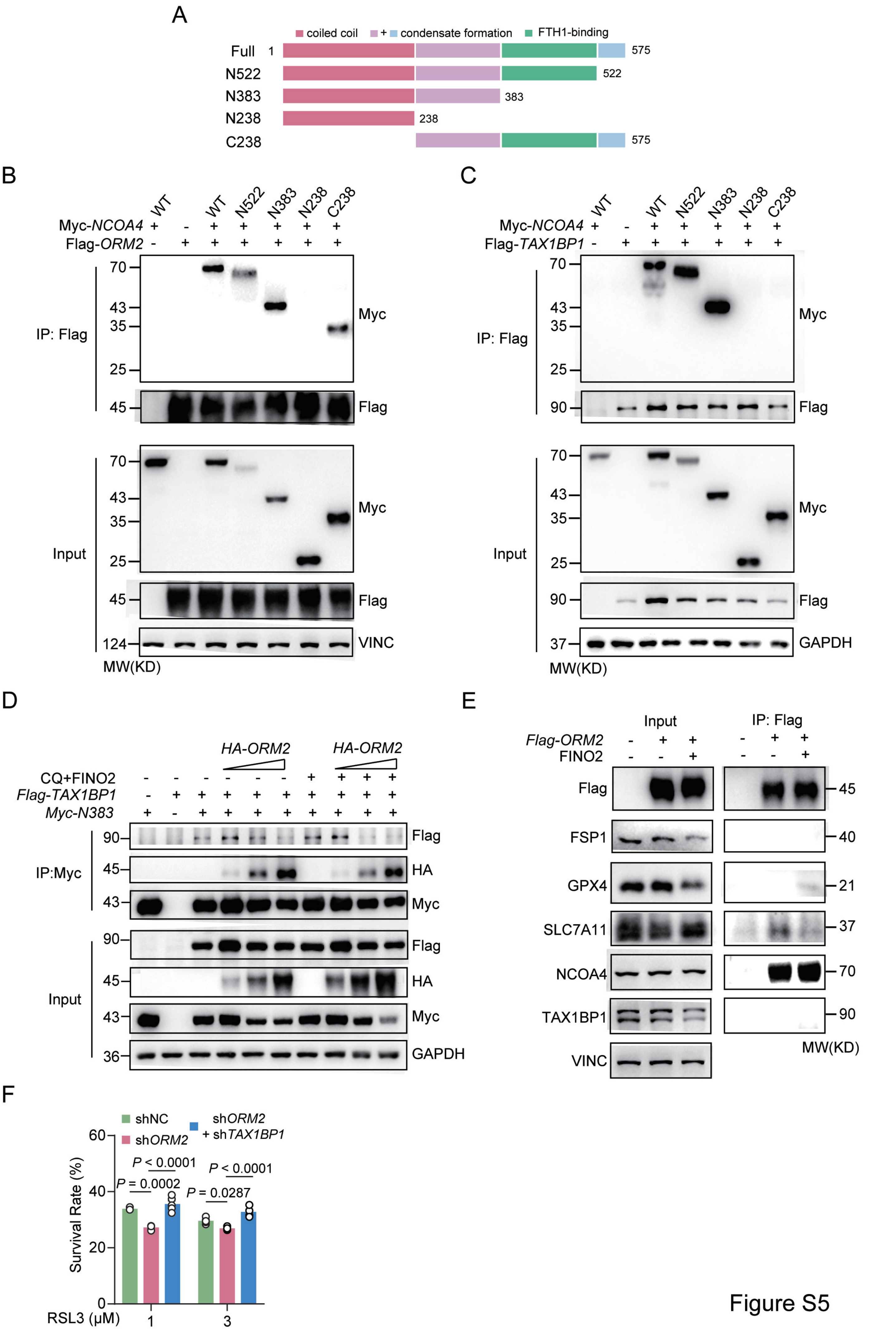
A
coiled coil
+ condensate formation
FTH1-binding
Full 1 575
N522 522
N383 383
N238 238
C238 575
B
WT - WT N522 N383 N238 C238
Myc-NCOA4 + - + + + + +
Flag-ORM2 - + + + + + +
IP: Flag
Myc
Flag
Input
VINC
70
43
35
25
45
124
MW(KD)
C
Myc-NCOA4
Flag-TAX1BP1
GAPDH
90
37
D
HA-ORM2
CQ+FINO2
Flag-TAX1BP1
Myc-N383
IP:Myc
HA
36
E
Input
IP: Flag
Flag-ORM2
FINO2
FSP1
GPX4
SLC7A11
NCOA4
TAX1BP1
40
21
F
shNC
shORM2
shORM2 + shTAX1BP1
Survival Rate (%)
P < 0.0001
P = 0.0002
P = 0.0287
RSL3 (μM)
1
3

Figure S5

Figure S5. ORM2 inhibits ferritinophagy by competitively binding to the N-terminus of NCOA4 (aa 1-383) with TAX1BP1

(A) Schematic diagram of NCOA4 variants used in this study.

(B) Interaction assays in HEK293T cells co-expressing *ORM2-Flag* and Myc-tagged full-length or truncated *NCOA4*. Lysates were immunoprecipitated with anti-Flag beads and analyzed by immunoblotting.

(C) Co-IP assays performed as in **(B)**, co-expressing the Myc-tagged *NCOA4* constructs with *TAX1BP1-Flag* to assess their interaction.

(D) HEK293T cells were co-transfected with *Flag-TAX1BP1*, the N383 truncation of *Myc-NCOA4*, and increasing amounts of *HA-ORM2* plasmids. Cells were then treated with 8 μM FINO2 plus 10 μM chloroquine (CQ) or DMSO vehicle. Co-IP were performed using Myc beads, and Flag, HA, Myc, and GAPDH antibodies were used for IB.

(E) Lysates from HEK293T cells overexpressing *Flag-ORM2* or empty vector were immunoprecipitated using Anti-Flag agarose beads. Precipitated proteins and input lysates were analyzed by Western blot with antibodies against Flag, FSP1, GPX4, SLC7A11, NCOA4, and TAX1BP1.

(F) HepG2 cells with stable knockdown of *ORM2, TAX1BP1*, or both were treated overnight with the indicated concentrations of RSL3. Cell viability was assessed using the CCK8 assay.

Data are presented as mean ± SEM. In (F), data were analyzed by two-way ANOVA followed with Bonferroni's post hoc test. Exact *p* values are provided in the figure.

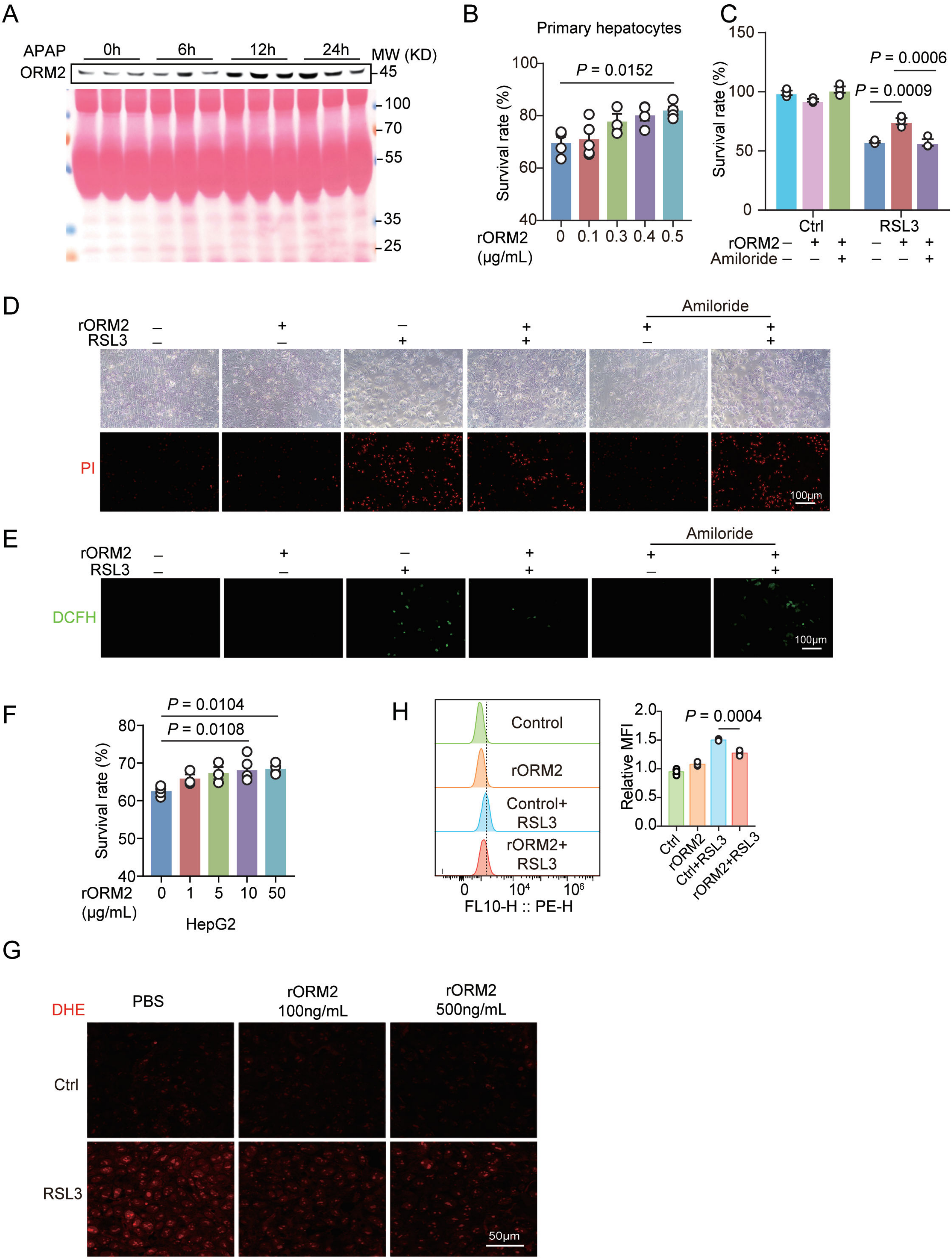
A
APAP
0h
6h
12h
24h
MW (KD)
ORM2
45
100
70
55
35
25
B
Primary hepatocytes
P = 0.0152
Survival rate (%)
rORM2 (μg/mL)
0 0.1 0.3 0.4 0.5
C
P = 0.0006
P = 0.0009
Survival rate (%)
Ctrl
RSL3
rORM2
Amiloride
D
rORM2
RSL3
Amiloride
PI
100μm
E
rORM2
RSL3
Amiloride
DCFH
100μm
F
P = 0.0104
P = 0.0108
Survival rate (%)
rORM2 (μg/mL)
0 1 5 10 50
HepG2
H
Control
rORM2
Control+ RSL3
rORM2+ RSL3
FL10-H :: PE-H
P = 0.0004
Relative MFI
Ctrl
rORM2
Ctrl+RSL3
rORM2+RSL3
G
DHE
PBS
rORM2 100ng/mL
rORM2 500ng/mL
Ctrl
RSL3
50μm

Figure S6

Figure S6. Infusion of recombinant ORM2 protein significantly alleviates acute liver injury

(A) Mice were treated with 300 mg/kg APAP, and serum ORM2 protein levels were assessed at indicated time points. ORM2 protein was detected by western blot, with total protein quantified using Ponceau S staining (n = 3 per group).

(B) Primary hepatocytes were pretreated with recombinant ORM2 (rORM2) protein at specified concentrations for 2 hours, followed by overnight treatment with 6 μM RSL3. Cell viability was assessed using the CCK8 assay.

(C) Primary hepatocytes were treated with Amiloride (2 μM) for 2 hours, then incubated with or without 500 ng/mL rORM2 for 2 hours, followed by 6 μM RSL3 for 12 hours. Cell viability was assessed using CCK8 assay.

(D) Cell death from **(C)** was measured by Propidium Iodide staining. Scale bars, 100 μm.

(E) ROS levels from **(C)** were measured by DCFH-DA staining. Scale bars, 100 μm.

(F) HepG2 cells were pretreated with recombinant ORM2 protein at specified concentrations for 2 hours, and then treated with 4 μM RSL3 for 12 hours, followed by CCK8 to assess cell viability.

(G) HepG2 cells were pretreated with recombinant ORM2 protein at specified concentrations for 2 hours, and then treated with 4 μM RSL3 for 4 hours, followed by DHE staining to assess ROS levels. Scale bars, 50 μm.

(H) HepG2 cells were incubated with 500 ng/mL recombinant ORM2 for 6 hours with or without 4 μM RSL3. FerrOrange (0.25 μM) staining was measured by flow cytometry.

All data in this figure are represented as mean ± SEM. In (B) and (F), data were

analyzed by one-way ANOVA with Dunnett's test; the remaining data were analyzed by two-way ANOVA followed with Bonferroni's post hoc test. Exact *p* values are provided in the figure. "n" refers to biological replicates. All experiments were performed in triplicates.

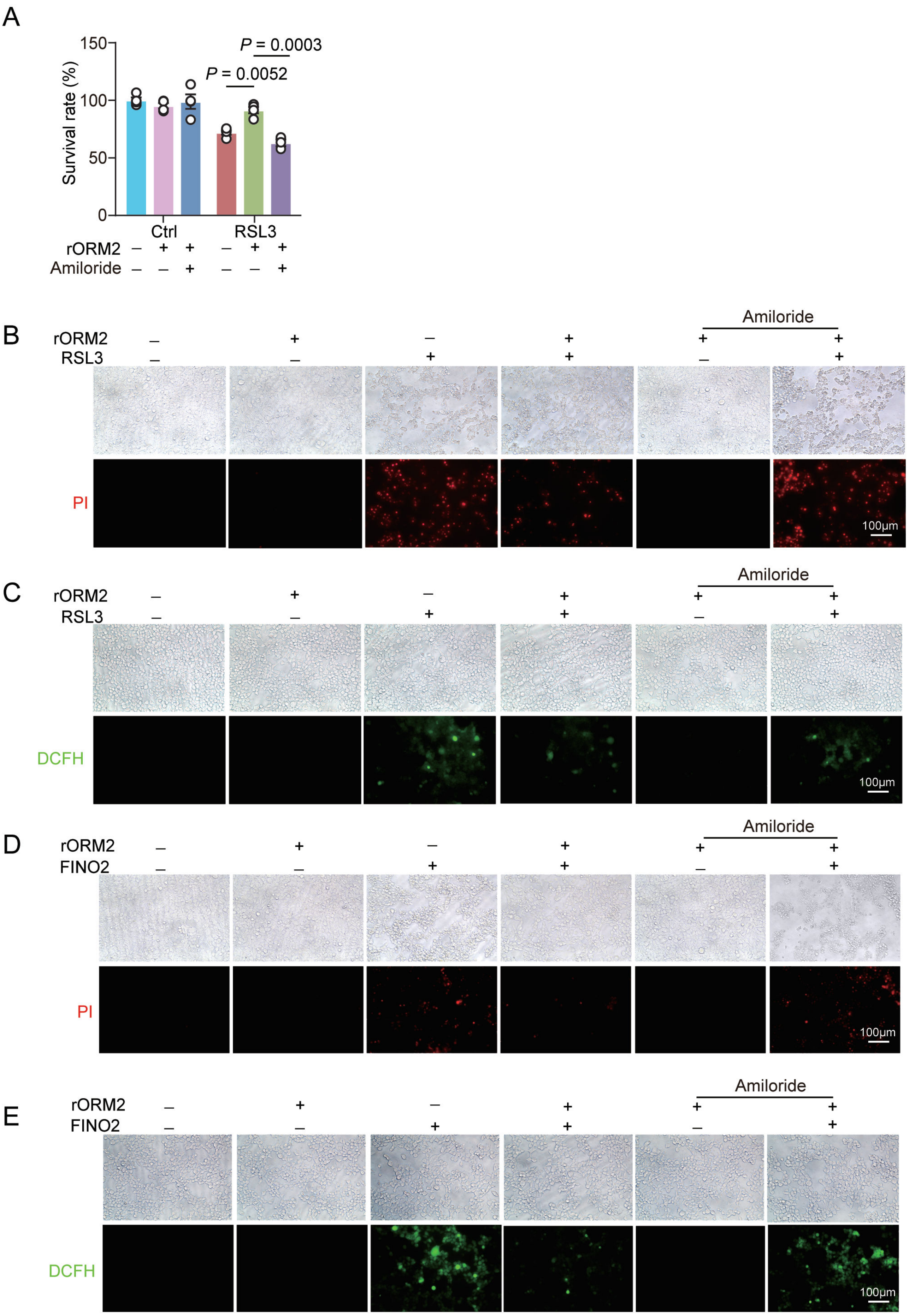
A
150
100
50
0
Survival rate (%)
P = 0.0003
P = 0.0052
Ctrl
RSL3
rORM2
Amiloride
B
rORM2
RSL3
Amiloride
PI
100μm
C
rORM2
RSL3
Amiloride
DCFH
100μm
D
rORM2
FINO2
Amiloride
PI
100μm
E
rORM2
FINO2
Amiloride
DCFH
100μm

Figure S7

Figure S7. The protective role of recombinant ORM2 protein against ferroptosis in renal cells

(A-C) HEK293T cells were treated with 2 μM Amiloride for 2 hours, then incubated with or without 1 μg/mL rORM2 for 2 hours. Cells were then exposed to 1 μM RSL3 for 12 hours. Cell viability was assessed using the CCK8 assay **(A)** and Propidium Iodide staining **(B)** and ROS levels were measured by DCFH-DA staining **(C)**. Scale bars, 100 μm.

(D-E) HEK293T cells were treated with 2 μM Amiloride for 2 hours, then incubated with or without 1 μg/mL rORM2 for 2 hours. Cells were then exposed to 8 μM FINO2 for 12 hours. Cell viability was assessed using Propidium Iodide staining **(D)** and ROS levels were measured by DCFH-DA staining **(E)**. Scale bars, 100 μm.

All data in this figure are represented as mean ± SEM. In (A), data were analyzed by two-way ANOVA followed with Bonferroni's post hoc test. Exact *p* values are provided in the figure. All experiments were performed in triplicates.

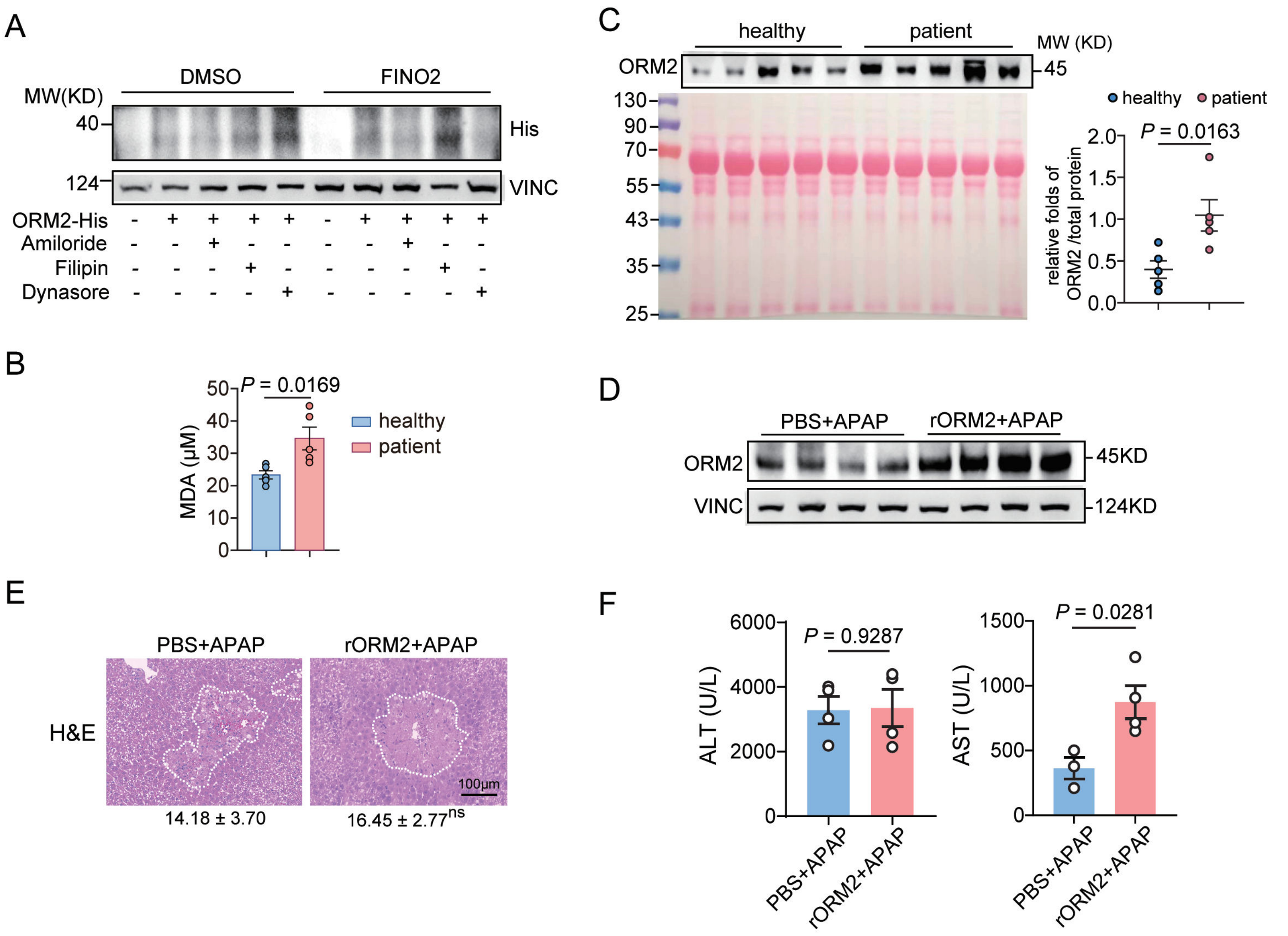
A
DMSO
FINO2
MW(KD)
40
His
124
VINC
ORM2-His
Amiloride
Filipin
Dynasore
B
P = 0.0169
MDA (μM)
healthy
patient
C
healthy
patient
MW (KD)
ORM2
45
healthy
patient
P = 0.0163
relative folds of ORM2 /total protein
D
PBS+APAP
rORM2+APAP
ORM2
45KD
VINC
124KD
E
PBS+APAP
rORM2+APAP
H&E
100μm
14.18 ± 3.70
16.45 ± 2.77ns
F
P = 0.9287
ALT (U/L)
P = 0.0281
AST (U/L)
PBS+APAP
rORM2+APAP

Figure S8

Figure S8. Functional analysis of recombinant ORM2 internalization and its effects in female mice

(A) 293T cells were pretreated for 1 h with the indicated endocytic inhibitors, followed by treatment with 1 μg/mL recombinant ORM2 protein alone or together with FINO2 for 6 h. Protein levels of ORM2 and GAPDH were analyzed by Western blot.

(B) Serum MDA levels in healthy volunteers (n = 5) and patients with acute liver or kidney injury (n = 5).

(C) ORM2 protein levels in serum samples from **(B)**. Left: representative Western blot of ORM2; Ponceau S staining shows total protein as loading control. Right: quantification of ORM2 levels normalized to total protein.

(D) Female mice were pre-treated with recombinant ORM2 protein (rORM2, 7 mg/kg) or PBS via intraperitoneal injection two hours prior to the administration of 300 mg/kg APAP; serum and liver tissues were collected 24 hours post-APAP challenge for subsequent analysis. Representative Western blot of ORM2 in liver tissues.

(E) Intrahepatic staining with quantification (below) of H&E from **(D)** was performed. Necrotic areas circled with white lines (n = 4 per group). Scale bar, 100 μm.

(F) Serum ALT and AST levels of mice in **(D)**.

All data in this figure are represented as mean ± SEM. In (B) and (F), data were analyzed by unpaired two-tailed Student's *t* test. Exact *p* values are provided in the figure, ns (no significance). All experiments were performed in triplicates.

Table S1. qPCR primers

gene	sequence (5'-3')
Mm-GAPDH	Forward: accacagtccatgccatcac
	Reverse: tccaccaccctgttgctgta
Mm-Cyp2e1	Forward: TGACTGACTGTCTCCTCATAGA
	Reverse: TCGGCCAAAGTCACAGAAATA
Mm-Gstp1	Forward: GCCCAGATGGATATGGTGAAT
	Reverse: GGCCTTCACGTAGTCATTCTTA
Mm-Gpx1	Forward: CGACATTGCCTGGAACTTTG
	Reverse: GGACAGCAGGGTTTCTATGT
Mm-Gpx2	Forward: CCAGCCCACCTTTAGTCTTAC
	Reverse: GTAGGGCAGCTTGTCTTTCA
Mm-Gpx4	Forward: CCGATATGCTGAGTGTGGTTTA
	Reverse: GGCTGCAAACTCCTTGATTTC
Mm-Gsr	Forward: CGGCCACTCCACTCATTATT
	Reverse: TGCAGGCCTTAACCTACTTTC
Mm-Cat	Forward: GATGGTAACTGGGATCTTGTGG
	Reverse: GTGGGTTTCTCTTCTGGCTATG
Mm-Sod1	Forward: CTCAGGAGAGCATTCCATCATT
	Reverse: CTCCCAGCATTTCCAGTCTT
Mm-Glrx	Forward: GCCAACATGTCTCTACCTCTAAG
	Reverse: GCTGACGAACAGTAGCAGAA
Mm-Prx1	Forward: GTTCTCACGGCTCTTTCTGT
	Reverse: GGGAGCAGGATACCCAATTT
Mm-Prx4	Forward: CTACCCACTGGATTTCACCTTT
	Reverse: GAGCATGCTACCACTTCAGTAT
Hs-GAPDH	Forward: CAAGAGCACAAGAGGAAGAGAG
	Reverse: CTACATGGCAACTGTGAGGAG
Hs-Orm2	Forward: GAGGCCGAGAACATGTTGCTCACC
	Reverse: TCGTCCAGGTAGGAACCAAACATC
Hs-FTH1	Forward: TACGCCTCCTACGTTTACCT
	Reverse: CTCTCCTCATGAGATTGGTGAAG
