# Supplementary material for: Hepatokine ORM2 suppresses pathological ferritinophagy to prevent acute tissue injury
Source: Cell Death Dis. 2026 Apr 30;17(1):578. doi: 10.1038/s41419-026-08803-0 (PMC13275894; doi:10.1038/s41419-026-08803-0)

Figure 1B:

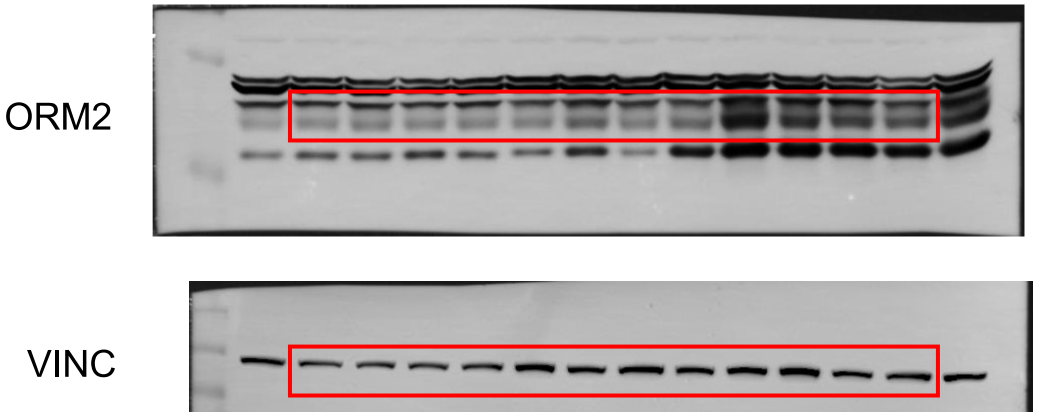

Figure 1G:

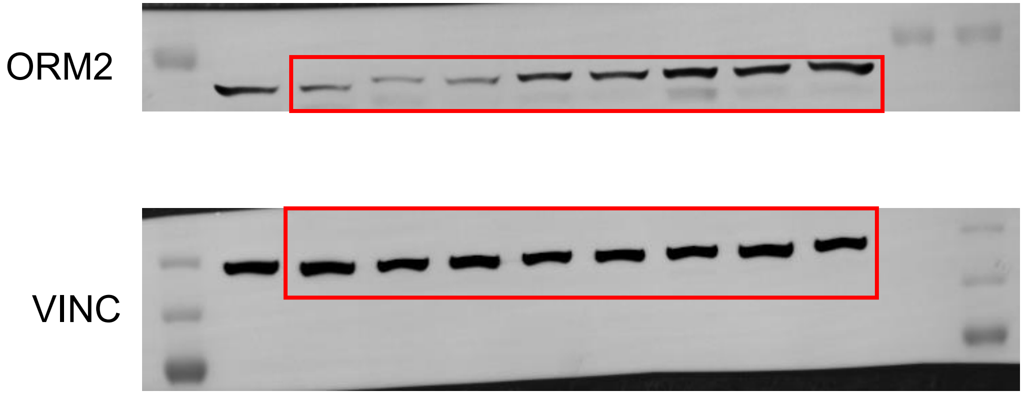

Figure 4A:

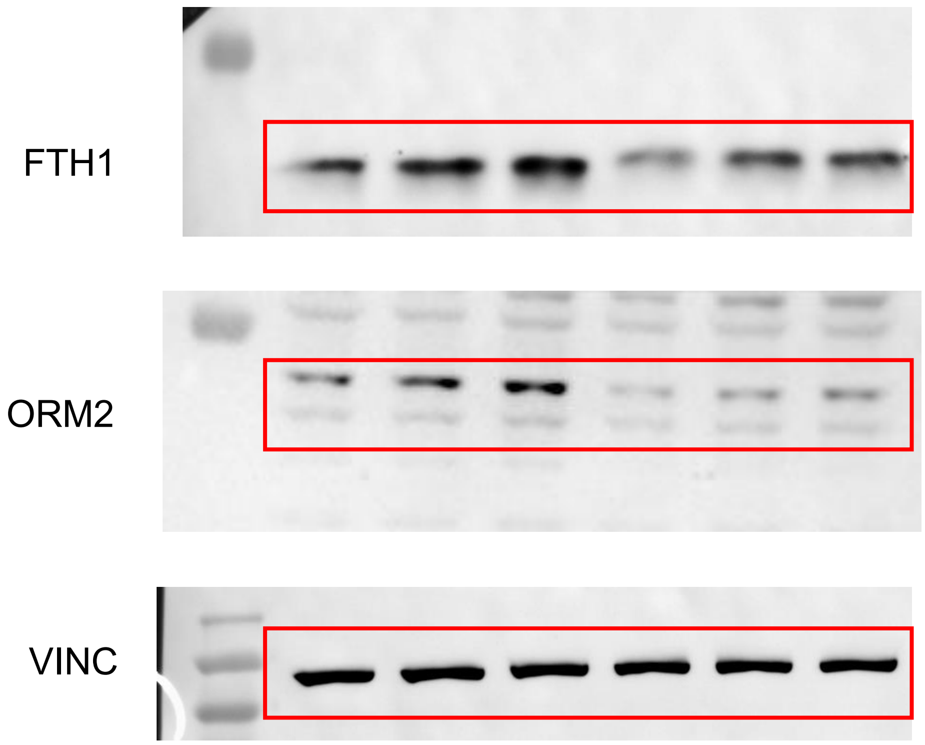

Figure 4C:

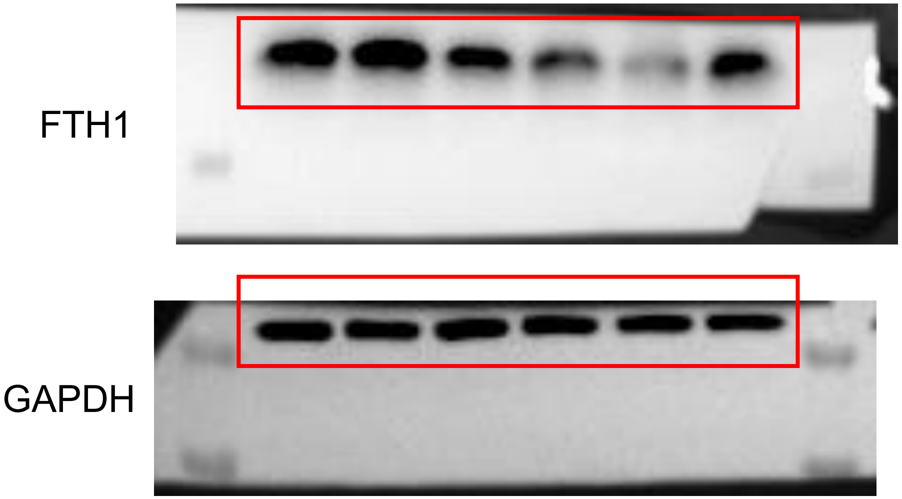

Figure 4E:

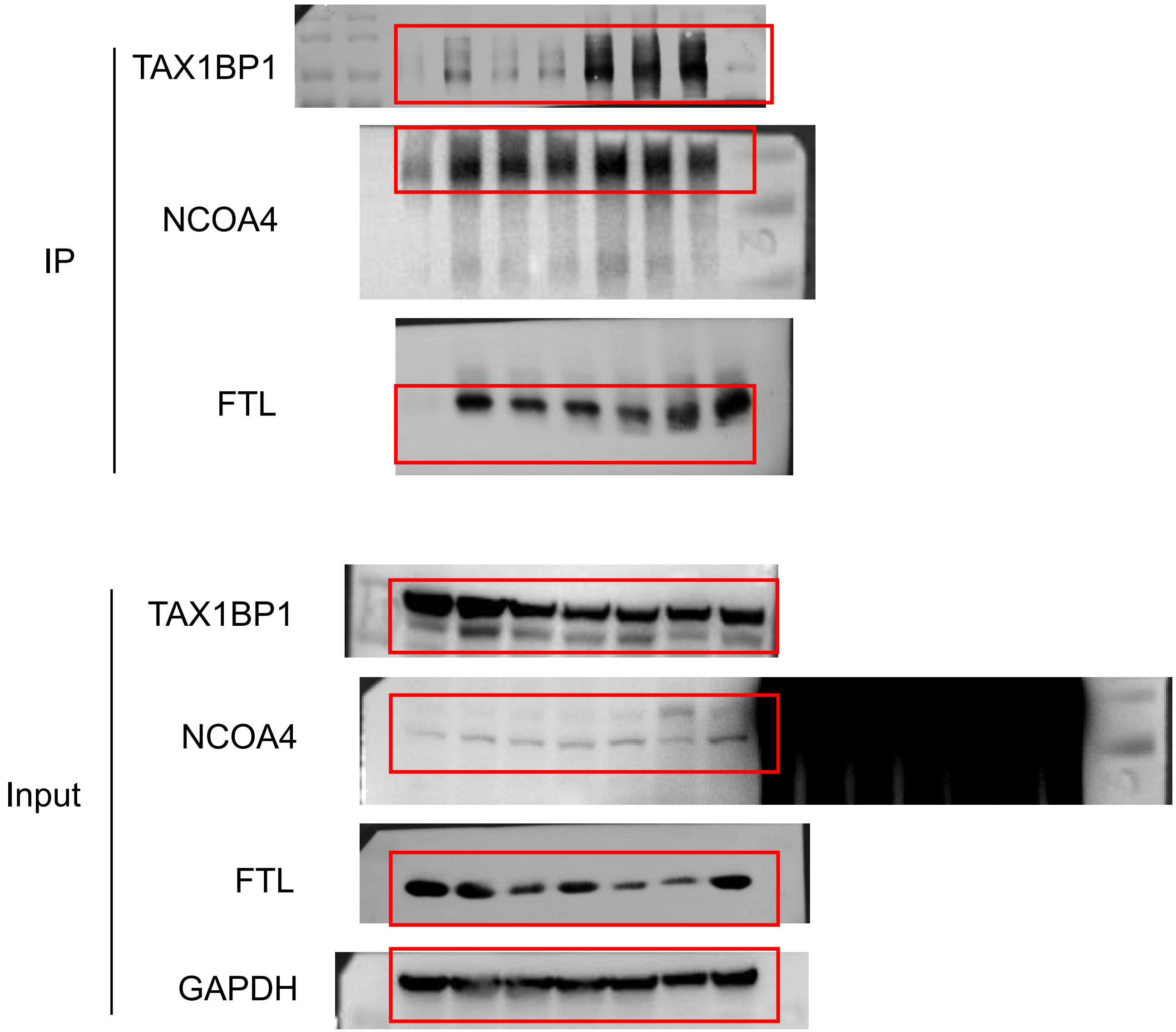

Figure 4F:

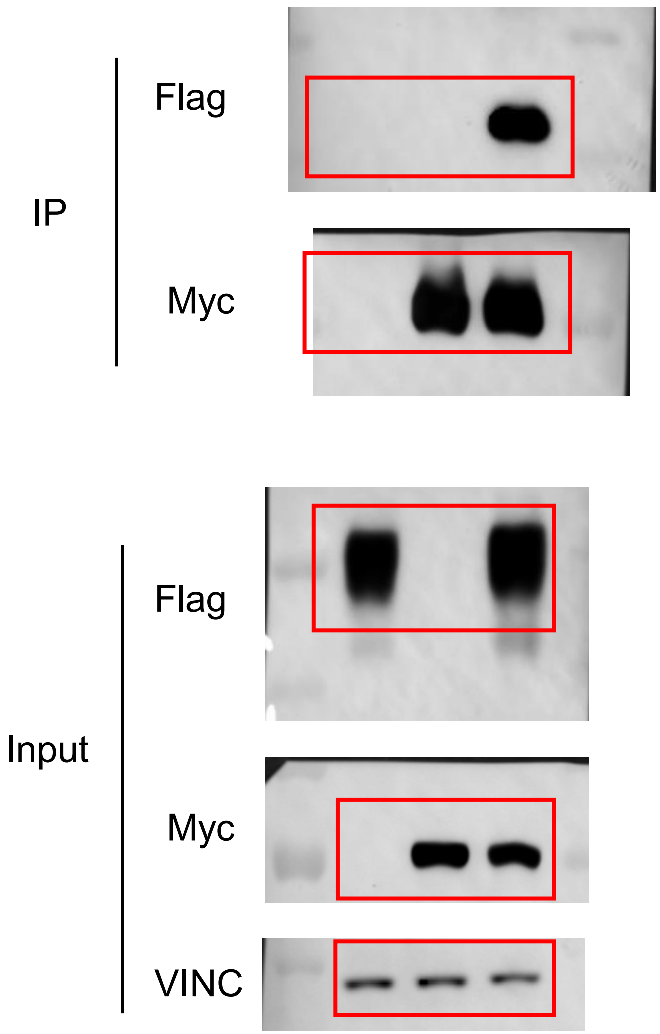

Figure 4G:

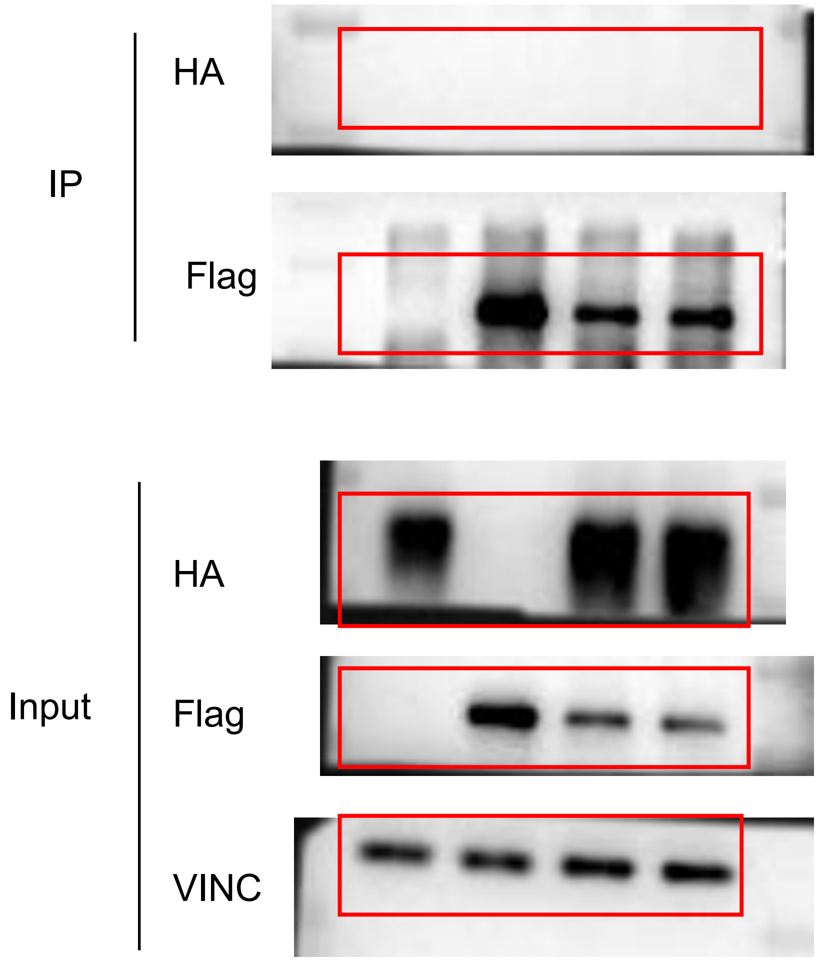

Figure 4I:

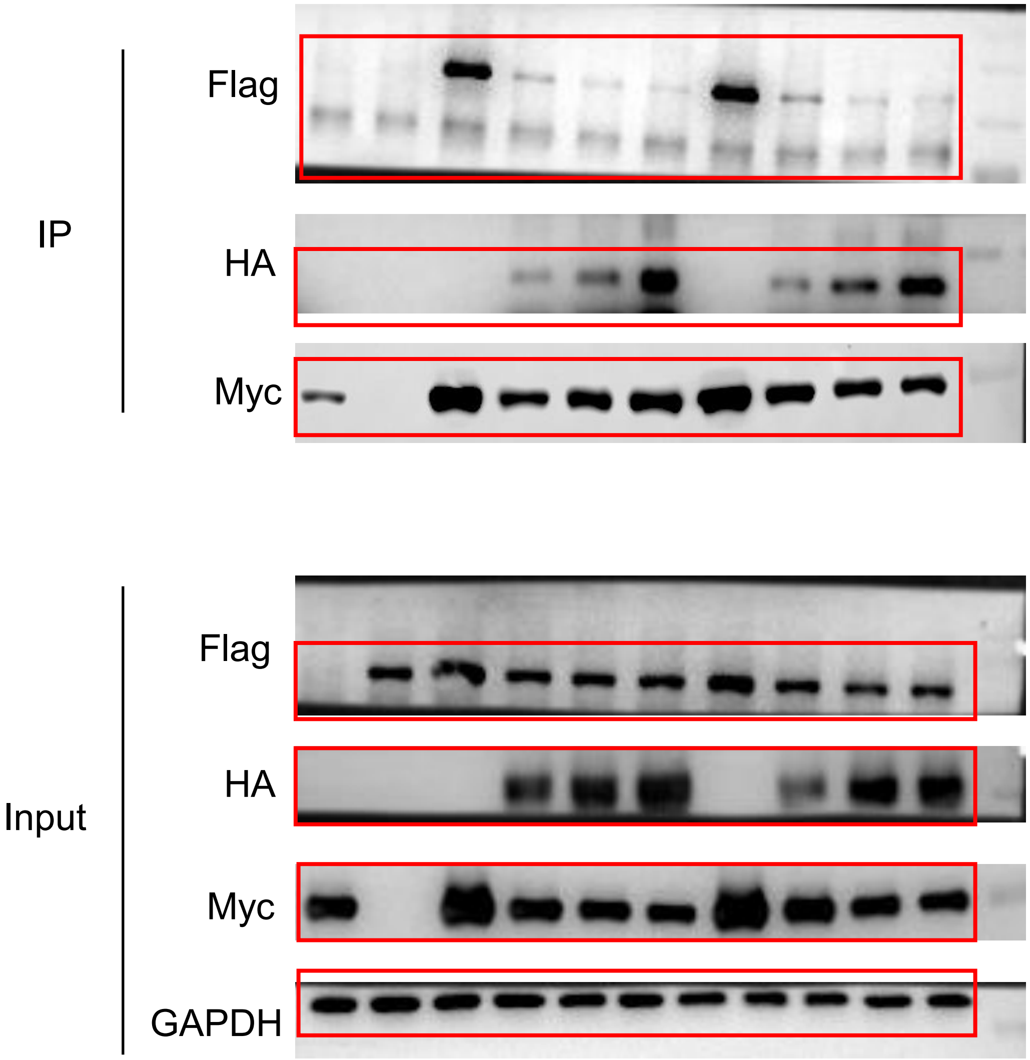

Figure 5A:

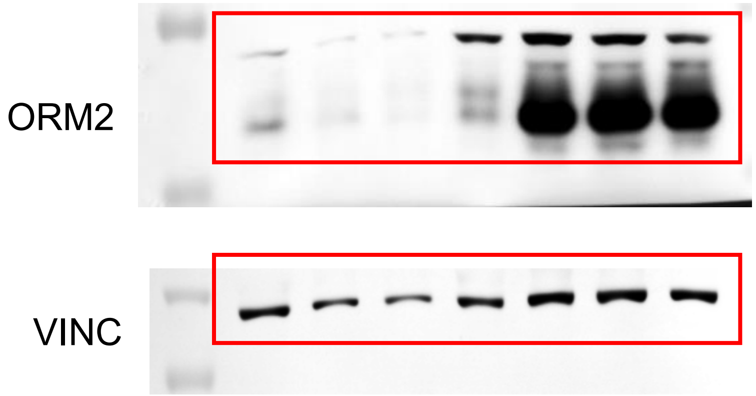

Figure 5E:

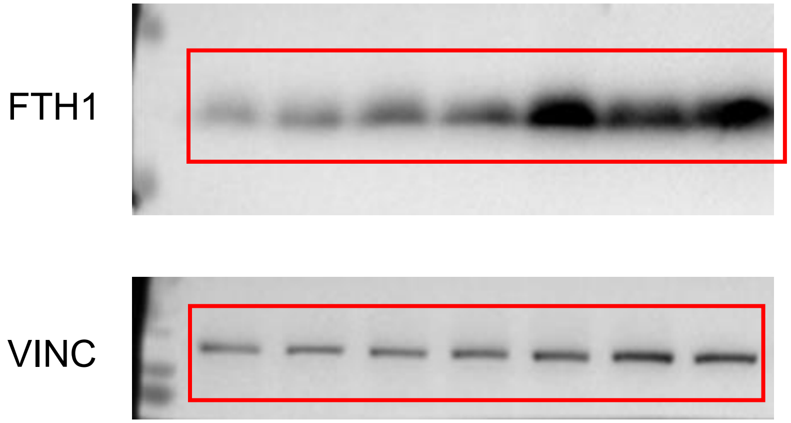

Figure 6A:

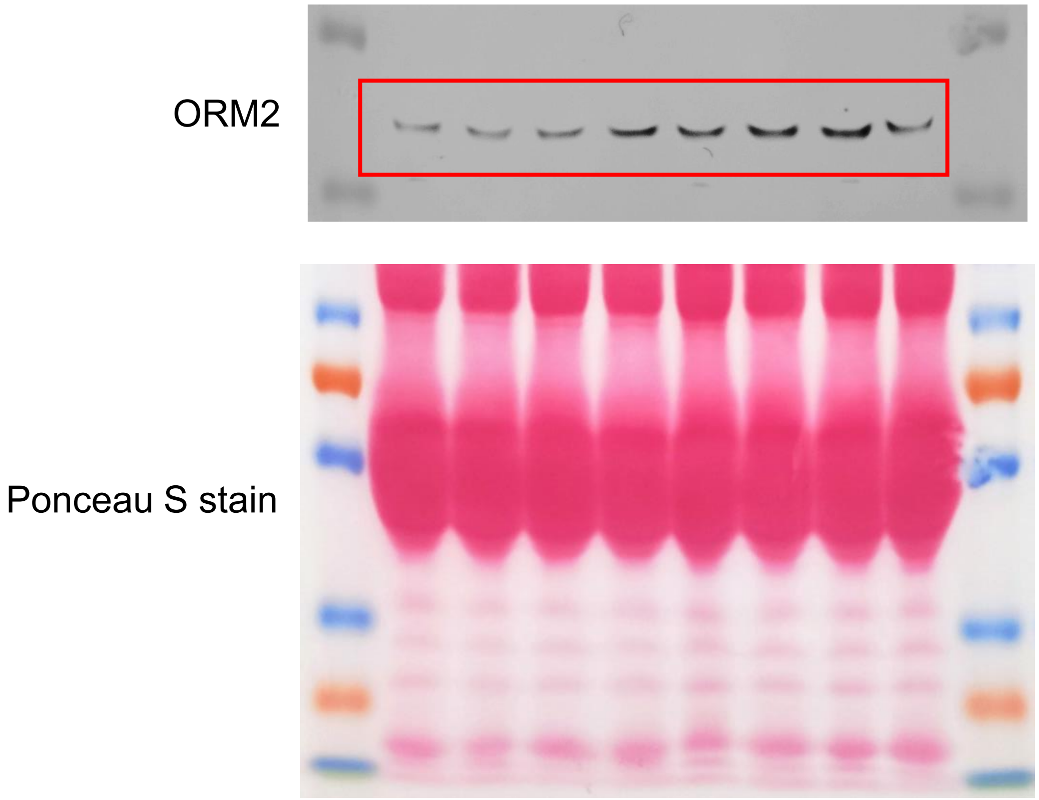

Figure 6B:

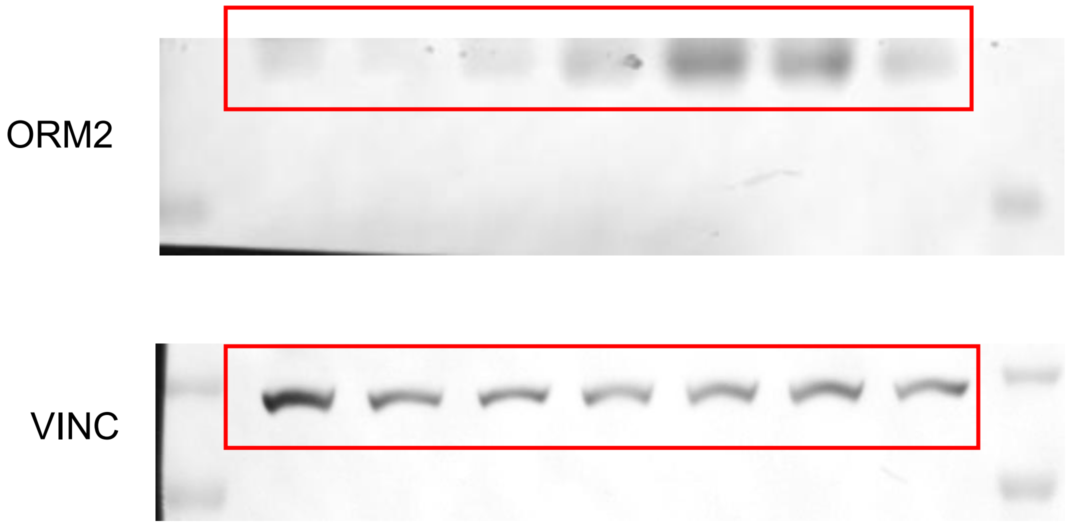

Figure 7F:

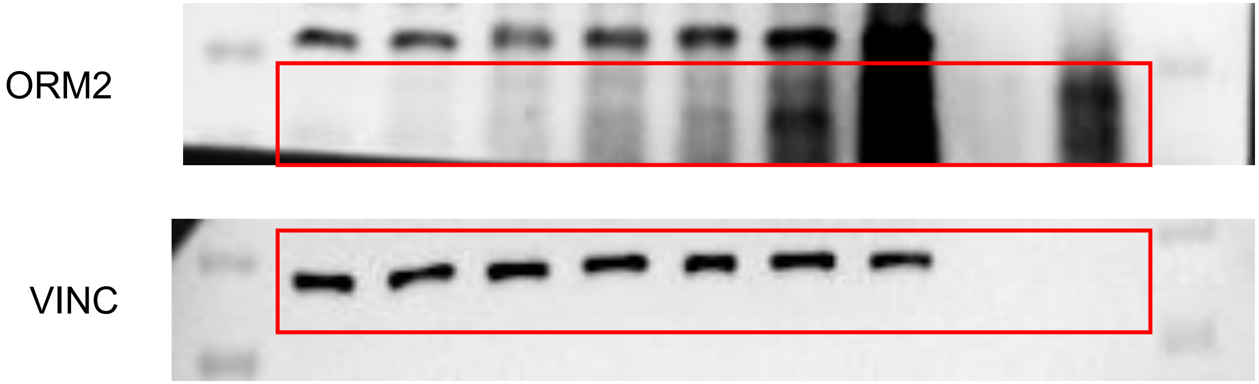

Figure 7I:

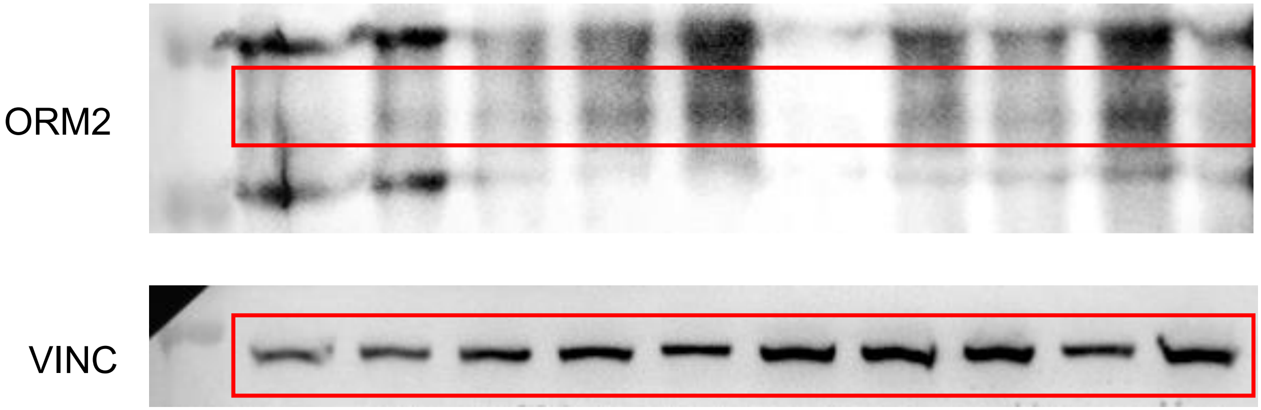

Figure S2A:

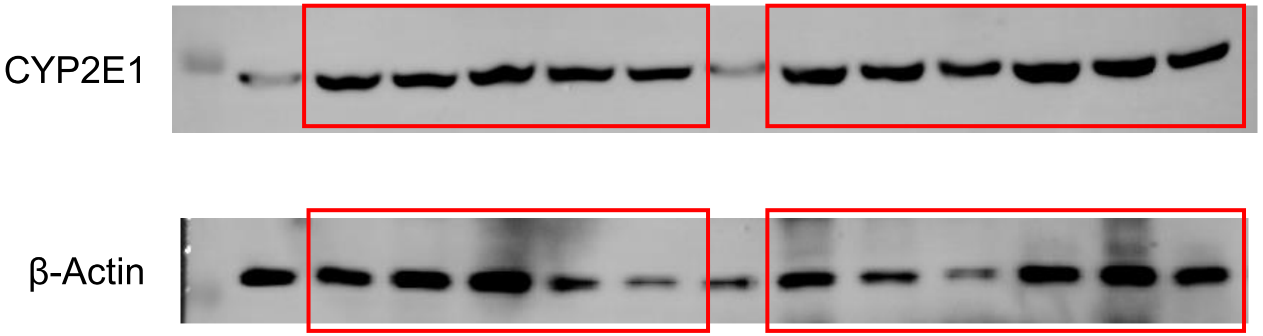

Figure S2G:

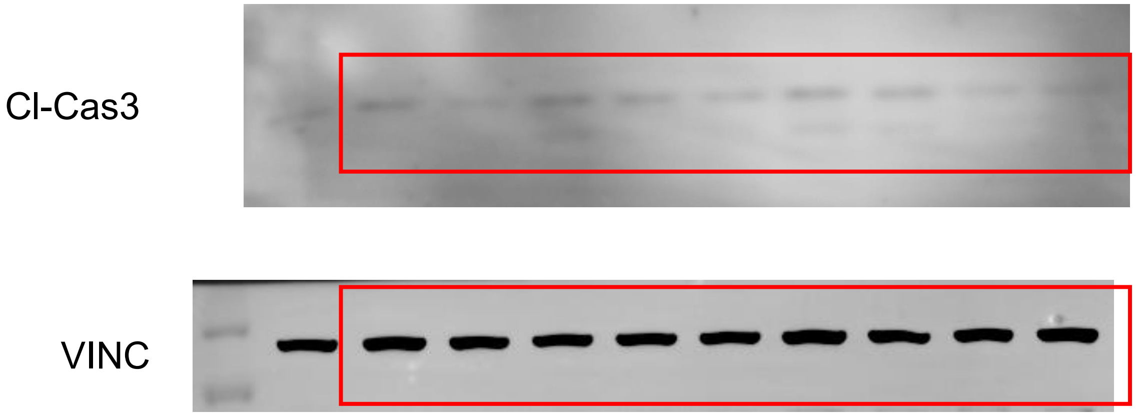

Figure S2I:

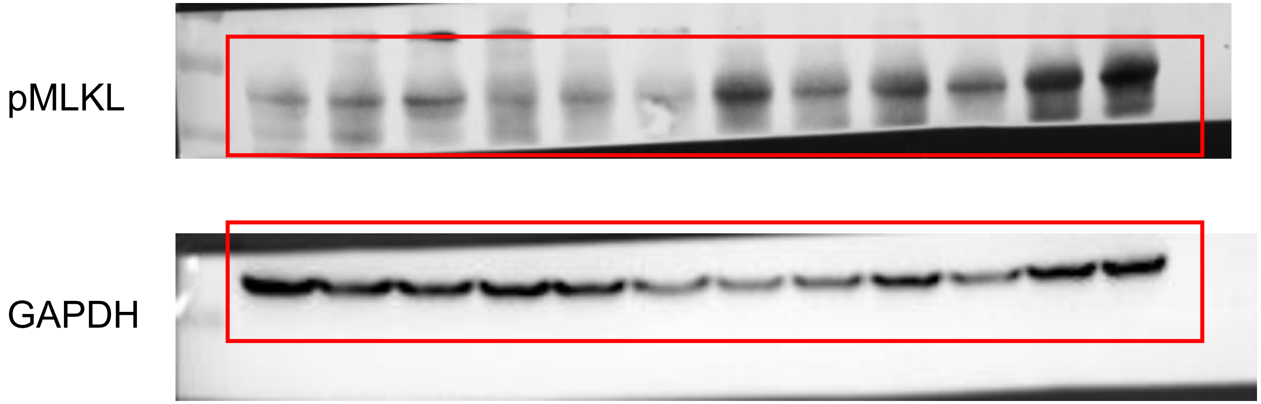

Figure S2K:

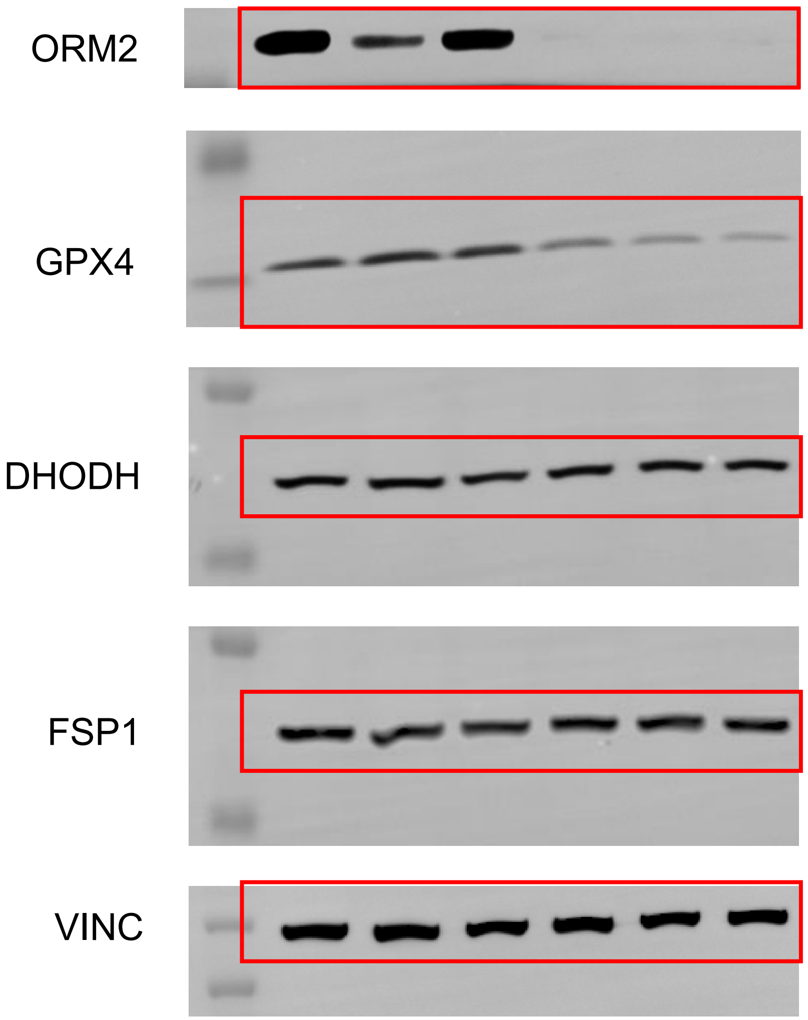

Figure S3C:

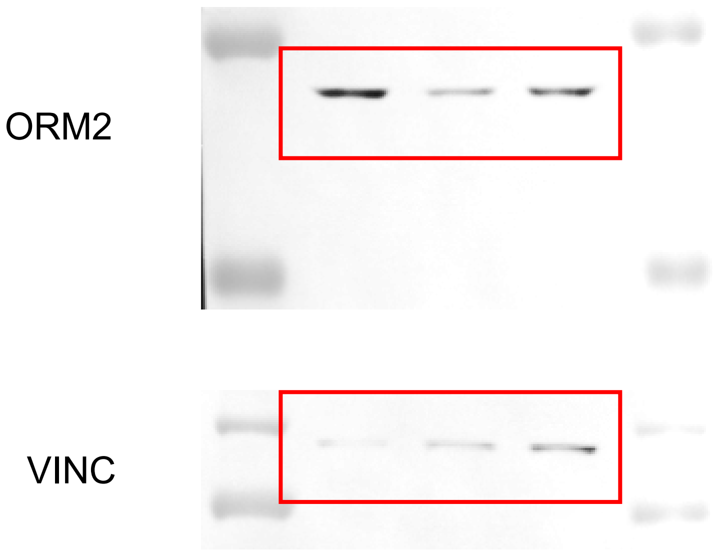

Figure S4A:

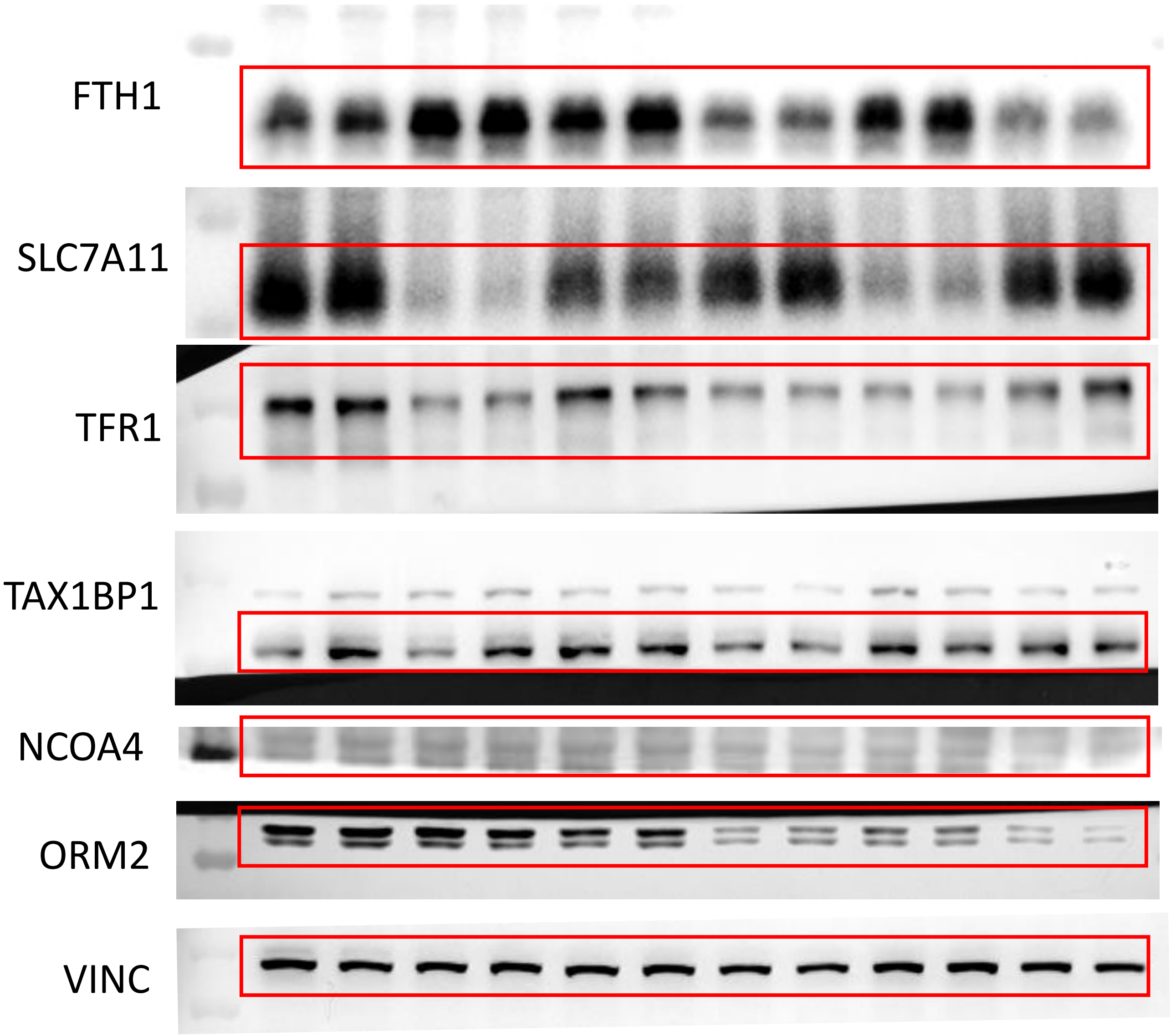

Figure S4B:

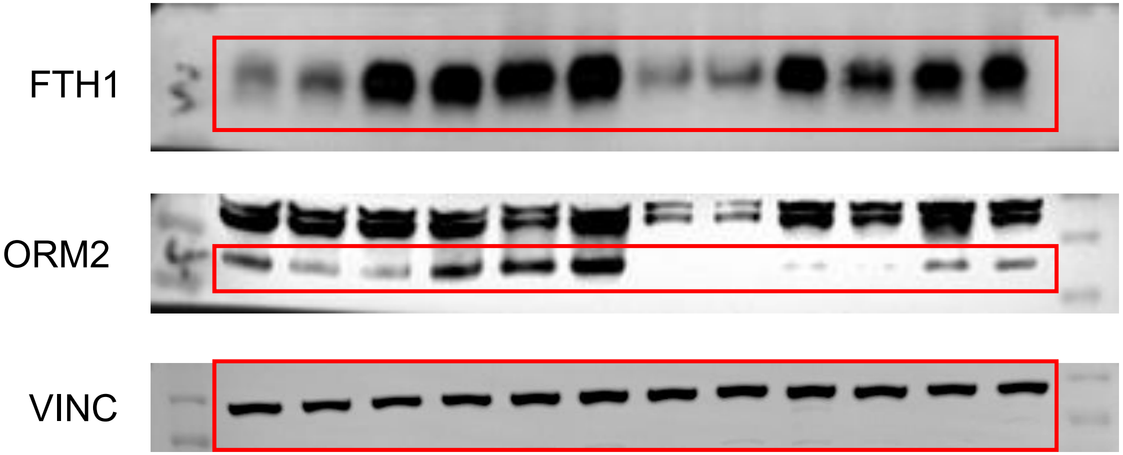

Figure S5A:

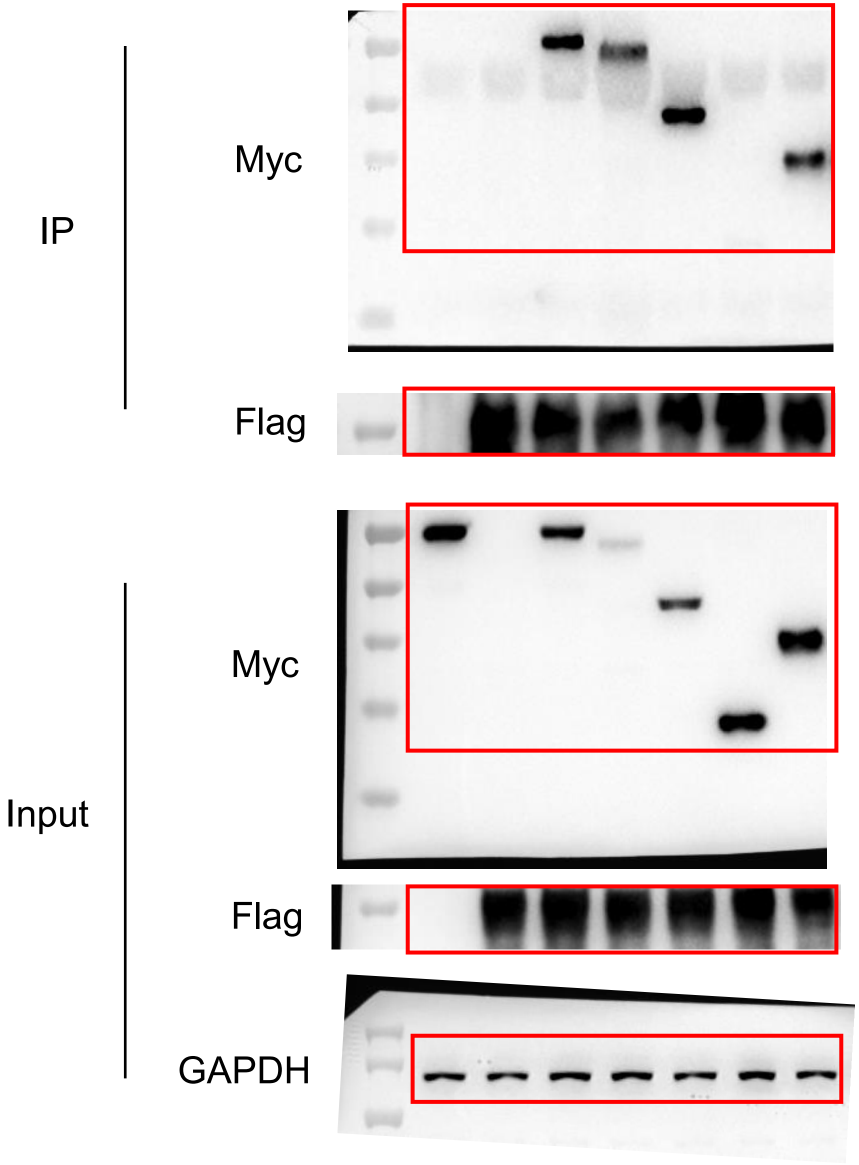

Figure S5B:

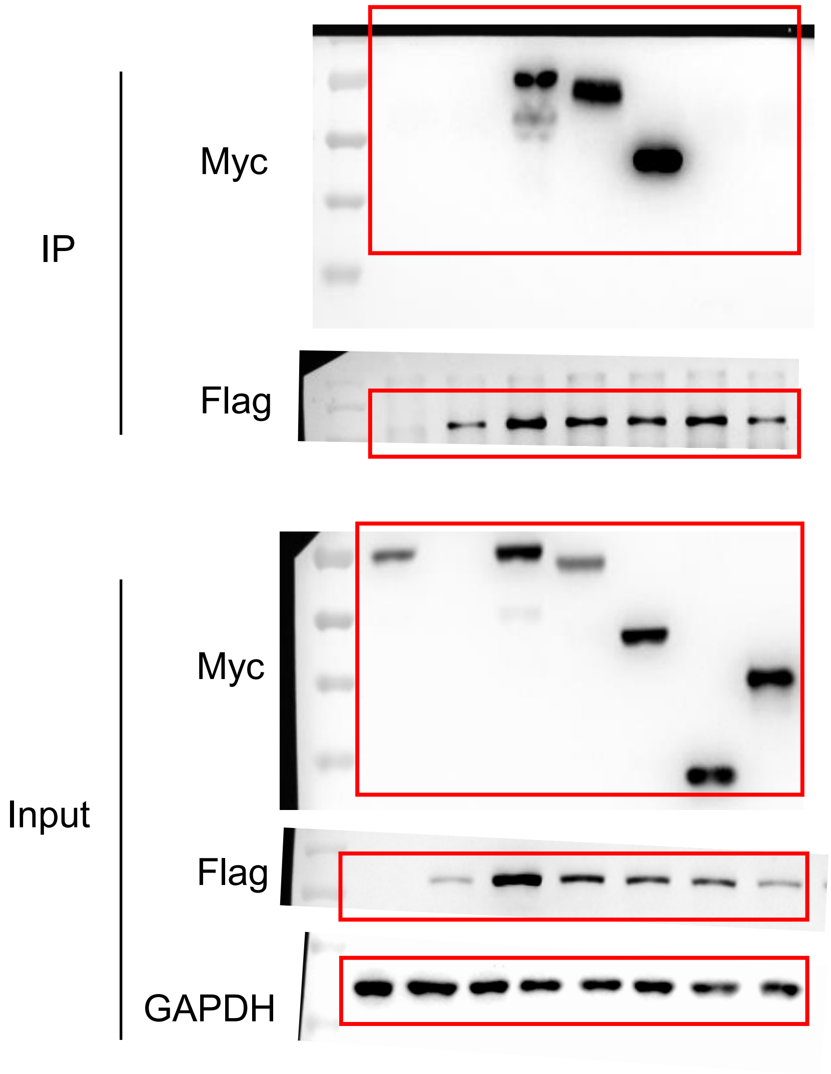

Figure S5D:

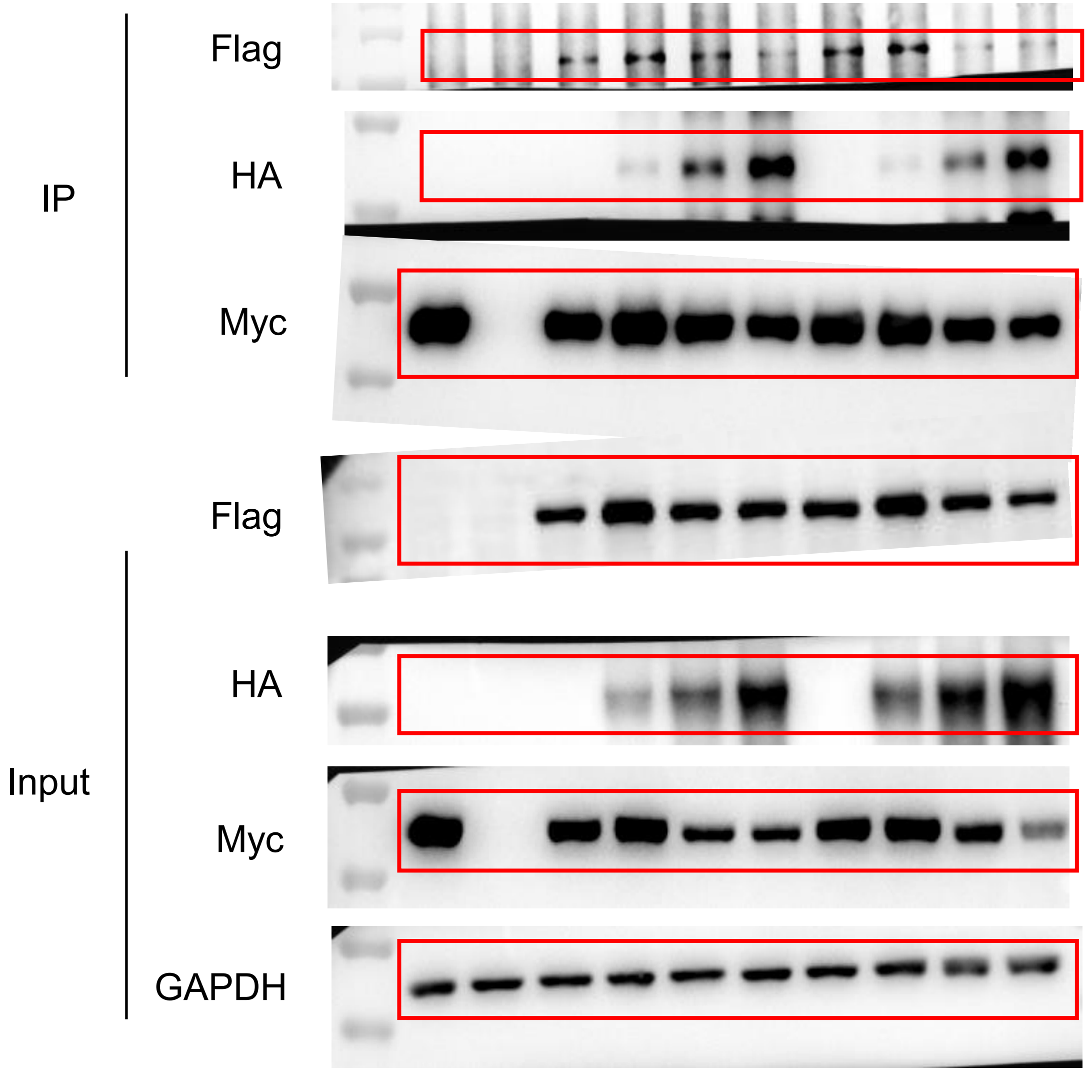

Figure S5E:

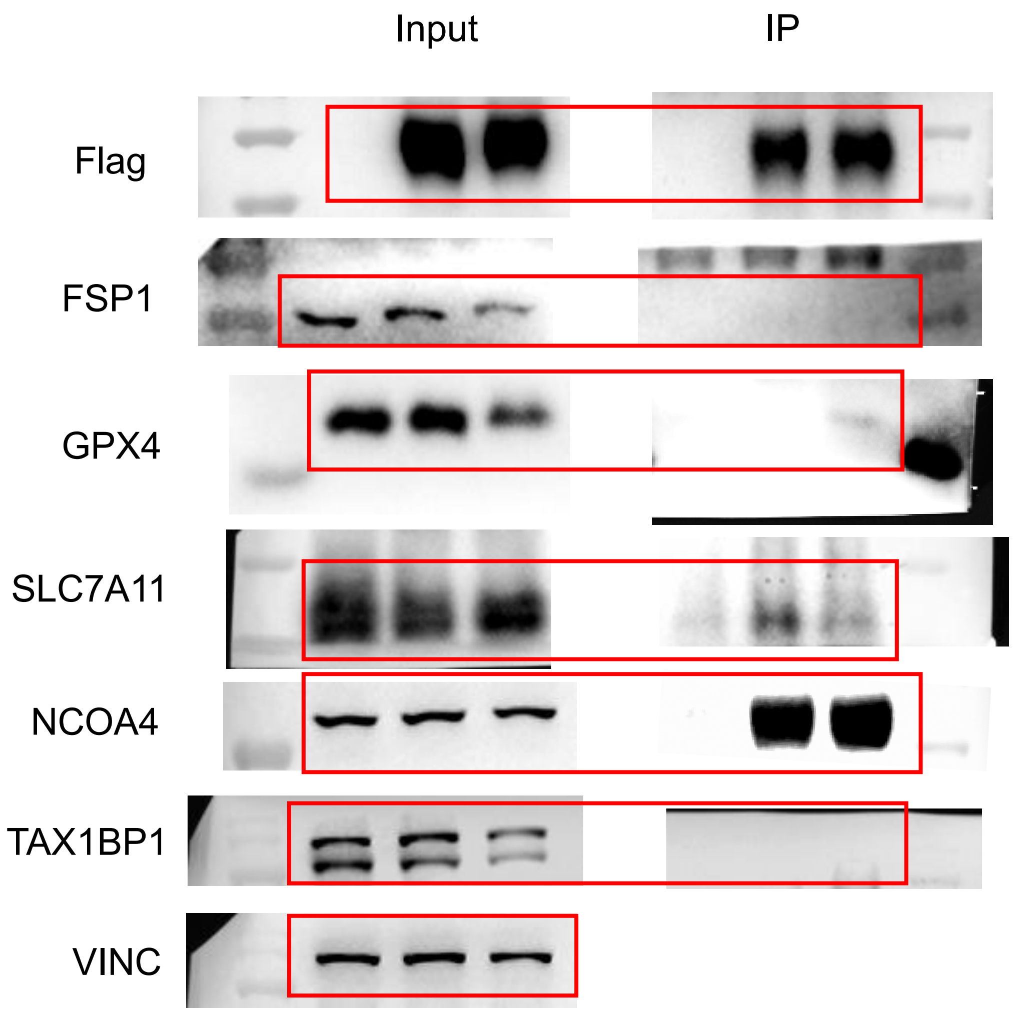

Figure S6A:

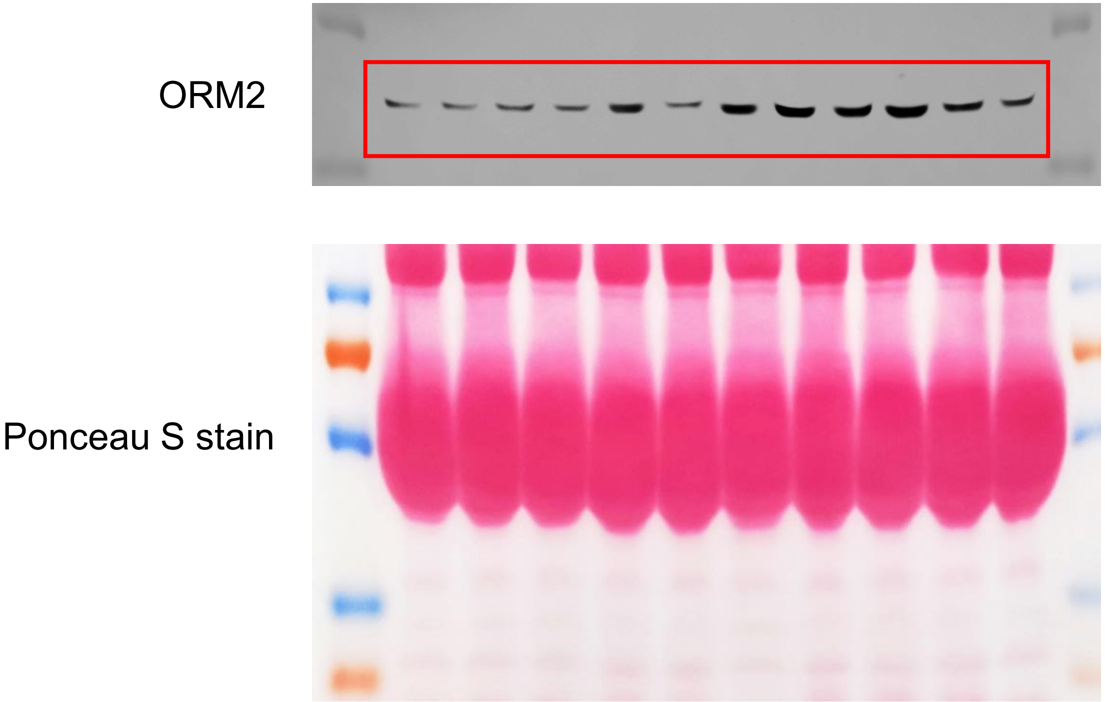

Figure S8C:

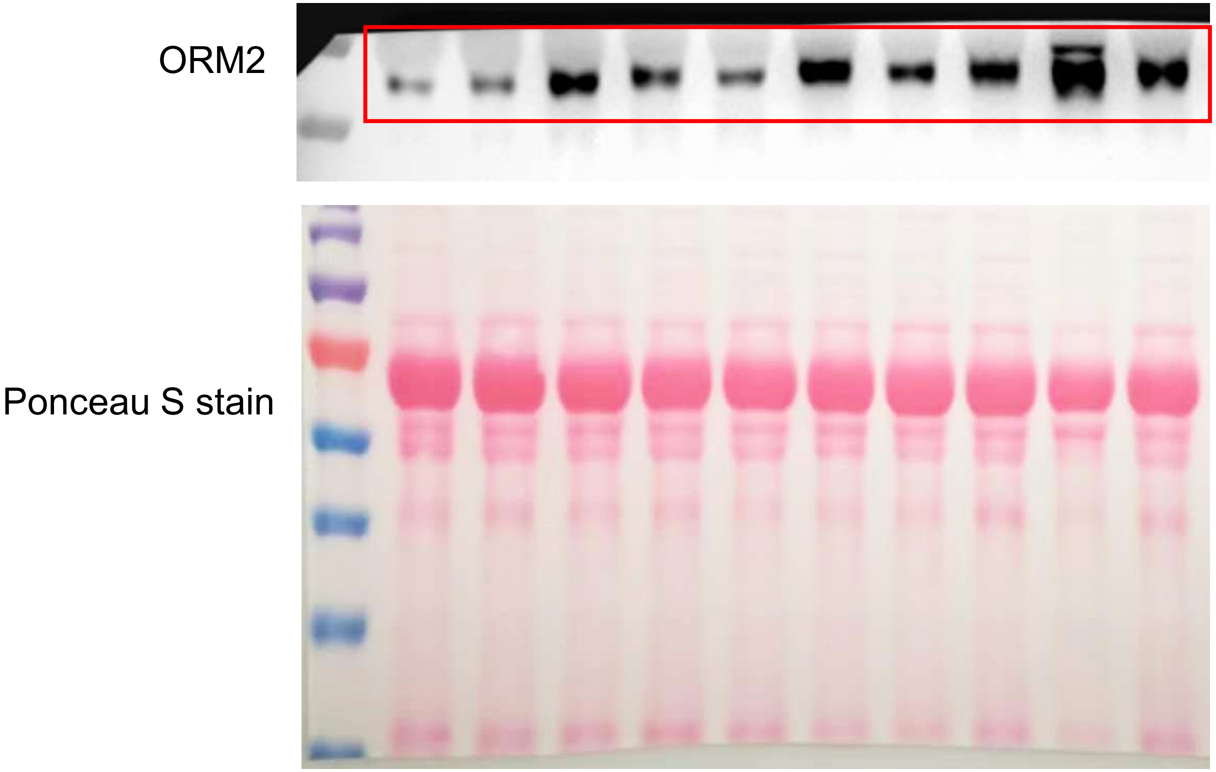

Figure S8D:

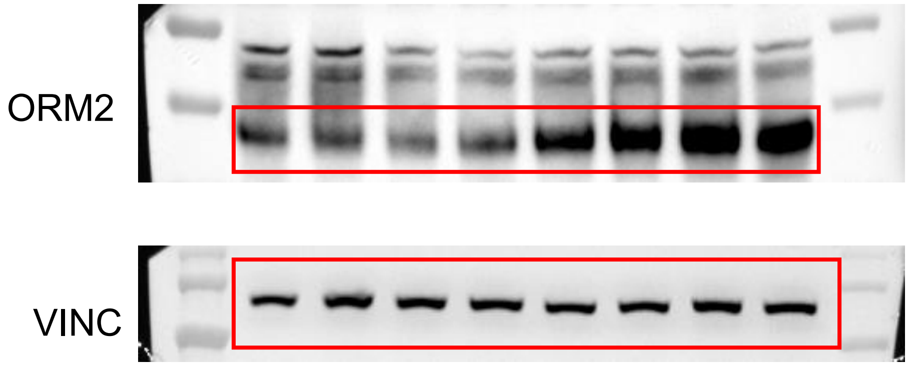

Supplement: Supplementary file 2 — original western blots [file 41419_2026_8803_MOESM2_ESM.pdf]
